# Microbiome Shifts in Peri-Implantitis: Longitudinal Characterization of Dysbiosis and Resolution

**DOI:** 10.1016/j.identj.2025.100951

**Published:** 2025-08-13

**Authors:** Songtham Anuntakarun, Sermporn Thaweesapphithak, Annop Krasaesin, Sasiprapa Prommanee, Sirikarn Arunyanak, Kajorn Kungsadalpipob, Aneesha Acharya, Thantrira Porntaveetus, Nikos Mattheos

**Affiliations:** a Center of Excellence in Precision Medicine and Digital Health, Department of Physiology, Faculty of Dentistry, Chulalongkorn University, Bangkok, Thailand; b Program in Bioinformatics and Computational Biology, Graduate School, Chulalongkorn University, Bangkok, Thailand; c Clinical Research Center, Faculty of Dentistry, Chulalongkorn University, Bangkok, Thailand; d Center of Excellence in Periodontal Disease and Dental Implant, Department of Periodontology, Faculty of Dentistry, Chulalongkorn University, Bangkok, Thailand; e Department of Periodontics and Oral Implantology, Dr. D.Y. Patil Dental College and Hospital, Dr D Y Patil Vidyapeeth, Pune, India; f Periodontology and Implant Dentistry, Faculty of Dentistry, The University of Hong Kong, Hong Kong; g Geriatric Dentistry and Special Patients Care International Program, Faculty of Dentistry, Chulalongkorn University, Bangkok, Thailand; h Clinic of General-, Special Care and Geriatric Dentistry, Center for Dental Medicine, University of Zurich, Zurich, Switzerland.; i Department of Oral and Maxillofacial Surgery, Chulalongkorn University, Bangkok, Thailand; j Department of Dental Medicine, Karolinska Institute, Stockholm, Sweden

**Keywords:** Healthcare, Treatment, Implant, Microbiome, Sequencing, Health disparity

## Abstract

**Objective:**

To characterize the longitudinal dynamics of the peri-implant microbiome in patients with peri-implantitis and healthy implants.

**Methods:**

The peri-implant microbiome was characterized longitudinally in patients with peri-implantitis and healthy implants via 16S rRNA gene sequencing. Samples were collected at baseline, 3-, and 6-months post-treatment. Bioinformatic analysis was performed to identify significant microbial shifts over time

**Results:**

At baseline, peri-implantitis sites exhibited significantly higher relative abundances of *Prevotella* (8.44%) and *Fusobacterium* (16.91%), compared to healthy implant sites, which were dominated by *Streptococcus* (16.91%) and *Neisseria* (10.06%). Post-treatment, *Haemophilus* increased in both groups by 3 months, particularly in peri-implantitis sites (19.94%). At 6 months, *Streptococcus* increased in PI sites (13.10%), while *Veillonella* and *Neisseria* remained prevalent in healthy sites. Differential abundance analysis confirmed partial recovery of peri-implantitis sites, with baseline dysbiosis marked by increased *Veillonella, Treponema denticola*, and *Porphyromonas gingivalis.*

**Conclusions:**

This study highlights dynamic shifts in the peri-implant microbiome during peri-implantitis progression and recovery, marked by specific changes in *Prevotella, Fusobacterium, Haemophilus, Streptococcus, Veillonella,* and key periodontal pathogens. These longitudinal changes offer insights into disease pathogenesis and underscore the potential of microbiome-targeted therapies.

## Introduction

The oral microbiome refers to the community of microorganisms inhabiting the human oral cavity.^1^ The oral cavity provides 2 main types of surfaces where bacteria can grow: the hard surfaces of teeth and the soft tissues lining the mouth. These different surfaces allow various kinds of bacteria to attach and form communities, shaping the overall mix of microorganisms in the mouth.^2^ The diverse structures within the oral cavity, including the tongue, cheeks, gingival sulcus, teeth, tonsils, soft palate, and hard palate, provide multiple niches for bacterial community establishment.^3^ Next-generation sequencing (NGS) technologies have provided increasing insight into our understanding of the oral microbiome. Amplicon sequencing of the 16S rRNA gene has been widely used for oral microbiome taxonomic profiling.^4^, ^5^, ^6^

Peri-implantitis is a common plaque-induced inflammatory condition affecting patients with dental implants. Initiated as plaque-induced mucositis, the inflammation continues to affect deeper tissues marked by degeneration of collagen and gradual deterioration of the supporting bone structure.^7^ Although there is ample evidence to document the involvement of bacterial plaque in the initiation of inflammation, there is limited understanding regarding the mechanisms that underpin the transition from peri-implant mucositis transitions to peri-implantitis. Emerging paradigms for the understanding of peri-implantitis indicate a gradual and multi-stage establishment of a dysbiotic biofilm and a deregulated immune response, which finally results in bone tissue dysregulation.^8^ Thus, a longitudinal assessment of the bacterial community shifts in relation to the immune response can decipher the mechanisms leading to the clinical establishment of peri-implantitis.^9^ Next-generation sequencing data has revealed distinct microbial profiles associated with peri-implantitis, showing that Gram-negative, anaerobic organisms, including *Porphyromonas gingivalis, Tannerella forsythia*, and *Prevotella intermedia*, predominate in diseased peri-implant sites.^10^

Inter-individual variability in the microbiome is well recognized and necessitates longitudinal data to comprehensively understand dysbiotic shifts associated with implant health and disease. To this end, the present preliminary study employed a longitudinal, pre- and post-therapy with 16S rRNA sequencing analysis at 0, 3, and 6 months to investigate the temporal dynamics of the microbiome associated with peri-implantitis. This approach involved comparing peri-implantitis sites in 1 quadrant to healthy implant sites in another quadrant within the same individuals, thereby minimizing inter-individual variability and providing insights into microbial shifts induced by treatment.

## Materials and methods

### Study design

The study was approved by the Human Research Ethics Committee of the Faculty of Dentistry, Chulalongkorn University (HREC-DCU 2020-031) to conduct a prospective, observational cohort study at the Graduate Periodontics Clinic.

### Study subjects

The study included 42 samples from 7 subjects, each of whom had at least 1 implant diagnosed with peri-implantitis (PI) and at least 1 implant with clinically healthy peri-implant tissue (HI) at a minimum of 1-year after the prosthesis placement. The diagnosis of peri-implantitis and peri-implant health was based on the 2017 World Workshop in Periodontology case definitions.^11^ Peri-implant health was diagnosed based on no signs of inflammation, stable probing depth (PD), and no further bone loss after initial remodeling. Peri-implantitis was diagnosed if there was the presence of inflammation, increased PD, and progressive radiographic bone loss as compared to baseline defined at the connection of the prosthesis. Exclusion criteria included pregnancy or lactation, history of malignancy, autoimmune disease, or any debilitating systemic condition that would preclude participation, history of corticosteroid or anti-inflammatory drugs during the last 2 months, or any history of anti-cancer or immunosuppressant drug intake. All subjects provided written informed consent prior to participation in the study.

### Demographic and clinical data

Demographic data was recorded, including age, gender, smoking status, systemic diseases, history of past periodontitis, and current periodontal status based on the 2017 World Workshop in Periodontology.^12^ A periodontal examination was conducted for all subjects, assessing PD and BOP at 6 sites per implant.

### Treatment of peri-implantitis

The treatment sequence for peri-implantitis began with a non-surgical phase consisting of oral hygiene reinforcement, full-mouth oral prophylaxis, non-surgical biofilm debridement, and correction of any prosthetic plaque-retentive factors. All patients were required to receive periodontal treatment if needed prior to the surgical intervention. This was followed by a surgical phase, including access-flap or regenerative surgical therapy as indicated at the respective peri-implantitis sites. After these interventions, all subjects were enrolled in a supportive care program. All patients were recalled at 3 and 6 months, and the treatment outcomes were assessed at 6 months post-surgery.

### Sample collection and study design

Dental biofilm samples were collected from all implant patients at 2 sites: the disease group (PI), harvested from implant sites with peri-implantitis, and the control group (HI), representing implant sites without disease. Participants were instructed to avoid eating or drinking 3 hours before sample collection. Brushing was not permitted the night before and the morning of the collection day. Subgingival biofilm was collected using a sterile Gracey curette (Implacare™, Hu-Friedy Inc, USA) and pooled into 1.5 ml tubes. After collection, the samples were placed in a foam container with ice and immediately transported to the laboratory, and stored at -80°C for further extraction and analysis. This study employed a within-subject, paired-site sampling design to evaluate microbial changes following surgical treatment of peri-implantitis. Each participant contributed 1 diseased (PI) site and 1 contralateral or anatomically comparable healthy (HI) site. A total of 7 participants were enrolled including systemically healthy and medically controlled conditions. All participants had a history of periodontitis but were clinically stable and well-maintained at the time of sample collection, having completed non-surgical therapy prior to surgical intervention. Notably, the sample included 5 patients treated with regenerative surgical therapy using a Jason® membrane and Straumann® xenograft, and 2 patients who received access flap surgery.

### DNA extraction and 16S rRNA gene amplicon sequencing

DNA was extracted from the samples using the QIAamp DNA Microbiome Kit (Qiagen, Hilden, Germany) according to the manufacturer’s protocols. The extracted DNA was then subjected to 16S metagenomic amplicon sequencing, targeting the V3-V4 region of the 16S rRNA gene using the primers Bakt_341F (5′-CCTACGGGNGGCWGCAG-3′) and Bakt_805R (5′-GACTACHVGGGTATCTAATCC-3′). Sequencing was performed on the Illumina MiSeq platform with 300 bp paired-end reads (Macrogen, Seoul, South Korea), generating an output of approximately 100,000 reads per sample.

### Bioinformatics analysis and Statistical analysis

The samples in this study were analyzed using the DADA2 pipeline (version 1.18.0)^13^ in R (version 4.1.0). Chimera removal was performed using the remove Bimera Denovo function with default parameters. Taxonomy and species assignments were performed using the RDP naive Bayesian classifier method,^14^ based on the SILVA reference database (version 138.1).^15^ Downstream analyses were conducted using "Phyloseq," "Microbiome," "microbiome Marker," "metacoder," and, "MicoViz" packages.^16^, ^17^, ^18^, ^19^ Alpha diversity was calculated at the genus level using several indices: the Simpson index, which quantifies the probability that 2 randomly selected individuals belong to the same species and emphasizes dominant species; the Shannon index, which assesses species diversity by considering both abundance and evenness, reflecting community richness; and the Chao1 index, which estimates total species richness by extrapolating from observed data. Wilcoxon rank sum test was used to compare alpha diversity metrics between groups (*P = .*05). Beta diversity ordination plots were drawn using the Weighted Unifrac and Bray-Curtis distance metrics. Principal coordinate analysis (PCoA) was employed to visualize the clustering of the HI and PI groups.^20^ A nonparametric permutational multivariate analysis of variance (PERMANOVA)^21^ was used to measure the multivariate community-level differences between the groups. In addition, Ward’s clustering was applied to Bray-Curtis distances to cluster the samples based on microbiome species composition. Sankey plots were plotted to identify the trajectories of sites through the clusters over time. Chi square tests (*α* = 0.05) were employed to assess the association of cluster membership with site, patient, and time point of sample collection. Microbial taxa indicators of the clusters were identified using the Kruskal-Wallis test (Bonferroni adjusted *p*-value cut off = .001) with the microbiome Marker package.

A "core microbiome" was identified by selecting bacterial taxa that were present in at least 50% of samples within each group (HI and PI) considering all time points within each group separately (i.e., all time points in PI and all time points in HI), and exhibiting a minimum relative abundance of 0.1%.^22^ PICRUSt2 (Phylogenetic Investigation of Communities by Reconstruction of Unobserved States)^23^ was used to predict microbiome-associated pathways. PICRUSt2 utilizes 16S rRNA gene sequencing data to infer metagenomic pathway profiles by mapping the detected taxa to pathways available in the comprehensive MetaCyc database.^24^

Differences in clinical parameters between baseline and 6 months after treatment in the PI and HI groups were analyzed using the Wilcoxon signed rank test, with significance determined at a *p*-value < .05. Univariate testing for differential abundance analysis at species-level abundance patterns using the Differential abundance analysis was performed to evaluate species-level abundance patterns using the EdgeR method^25^ from the microbiome Marker R package.^18^ Differentially abundant species in HI vs. PI: were determined at 3 distinct time points: baseline, 3 months post-treatment, and 6 months post-treatment. Temporal within-group comparisons in HI and PI assessed changes in species abundance by comparing baseline to 3 months post-treatment, baseline to 6 months post-treatment, and 3 months to 6 months post-treatment. Multivariate differential abundance analysis was done using MaAslin2^26^ using a mixed-effects model with the subject as a random effect, sampling time point and disease status (PI/HI) as fixed effects to identify differentially abundant species after catering for subject-level clustering.

## Results

### Demographic data

**Fig. 1:**
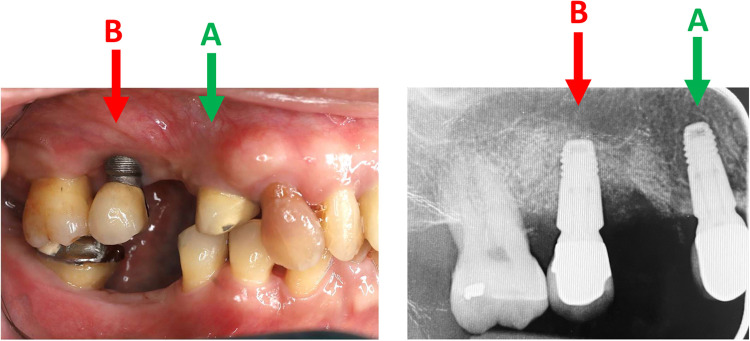
Clinical presentations of peri-implant tissue health and disease. Clinical photograph of a healthy implant (HI) site exhibiting normal peri-implant mucosal architecture, characterized by firm, pink tissue without signs of inflammation (A). Clinical photograph of a peri-implantitis (PI) site demonstrating clinical indicators of inflammation, including erythema, and swelling, with visible plaque accumulation around the implant (B).

All 7 participants (2 male-5 female) completed the 6-month study period. Hygienic phase was completed within 6-12 weeks, followed by removal of the prosthesis and surgery, which included an access flap, surface decontamination of the implants, and regeneration of the respective defects with guided bone regeneration with xenograft and a resorbable collagen membrane. The prosthesis was replaced after surgery in all cases. Participants had a mean age of 66.29 ± 12.6 years at the time of surgery. While 71.4% of participants had a history of periodontitis, 2 participants were diagnosed with diabetes and none of the participants were smokers. The location of the implant was mostly in the posterior region both in the PI and HI groups, while 4 of 7 implants in the PI group and 5 of 7 implants in the HI group were located in the maxilla. Clinical presentations of peri-implant tissue health and disease are illustrated in Figure 1.

### Clinical data: 6-month treatment outcomes

Treatment outcomes, 6 months after surgery, are presented in Table 1 with the changes from baseline. At 6 months, the mean ± SD probing depth of the PI and HI groups were 3.66 ± 1.14 mm and 2.52 ± 0.36 mm, respectively. The mean difference between before and after treatment in the PI group for the change in mean probing depth at 6 months post-surgery was 2.47 ± 1.65 mm (*P < .*01). The reduction in BOP was significantly observed in the PI group (*P < .*01). Absence of BOP was observed in 3 of 7 implants in the PI group and a reduction of BOP was observed in both PI and HI groups. Absence of inflammation (absence of BOP/SOP at all sites) was observed in 60% of both groups. No statistically significant differences in mean probing depth and BOP were found in the HI group.

**Table 1:** Treatment outcomes 6-month post-surgery (N = 7).

		Baseline	At 6-month after surgery	*P*-value
**Mean probing depth (mm ± SD)**	PI	6.14 ± 1.13	3.66 ± 1.14	.01^a^
	HI	2.77 ± 0.79	2.52 ± 0.36	NS
**Bleeding on probing**	PI	5.66 ± 0.51	1.71 ± 1.88	.004^a^
	HI	1.33 ± 1.96	1.73 ± 2.03	NS

a Wilcoxon signed rank test. NS, Not significant

### Microbial sequencing data

**Fig. 2:**
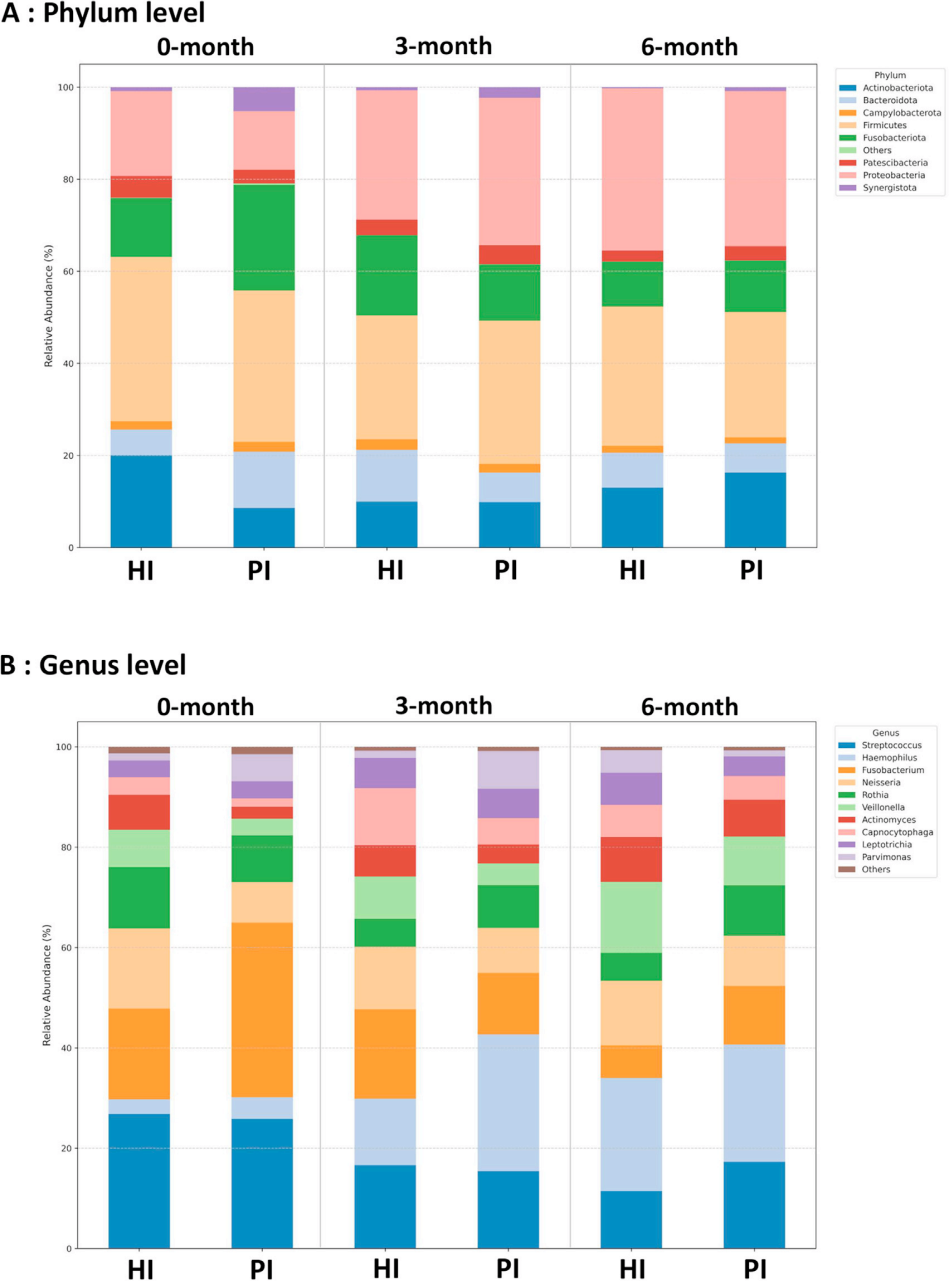
Microbial compositions in the 2 study groups. (A) Microbial composition at the genus level. (B) Microbial composition at the genus level at 0 months, 3 months, and 6 months. HI: Healthy Implants; PI: Peri-implantitis Implants.

A total of 42 biofilm samples yielded 3,754,071 raw reads. After quality filtering, 85% of the reads were retained for merging, resulting in 2,817,406 merged sequences. Chimera removal using the consensus method identified and excluded approximately 24% of the sequences as potential chimeras (Supplementary Table S1). From these sequences, 17 phyla, 65 orders, 116 families, 234 genera, and 358 species were identified. After excluding unassigned taxa, seventeen phyla were identified. Of those, the top 5 abundant phyla in PI and HI were Firmicutes, Proteobacteria, Actinobacteriota, Fusobacteriota and Bacteroidota. Phyla with an average abundance of less than 1 percent, including Chloroflexi, Cyanobacteria, Dadabacteria, Deinococcota, Desulfobacterota, Euryarchaeota, Planctomycetota, Spirochaetota, and Verrucomicrobiota, were grouped as the ‘Others’. Additionally, at 0 months, Firmicutes was the most abundant phylum in both groups, with a higher relative abundance in HI (35.49%) than in the PI group (32.14%). In contrast*,* Fusobacteriota was enriched in the PI group (22.48%) compared to the HI group (12.67%). At 3 months, Proteobacteria became the dominant phylum in both groups, with higher levels in the PI group (31.79%) than in the HI group (27.97%). Firmicutes remained slightly higher in the PI group than in the HI group. Actinobacteriota levels were similar in both groups, and Bacteroidota was lower in the PI group (6.38%) than in the HI group (11.18%). By 6 months, Proteobacteria remained dominant, with slightly higher levels in the HI group (35.11%) than in the PI group (33.43%). Firmicutes was more abundant in the HI group, while Fusobacteriota continued to decline, remaining higher in the PI group (Figure 2A).

The genus level analysis, the top 5 most abundant genera in the HI group were *Streptococcus* (12.68%), *Fusobacterium* (9.86%), *Neisseria* (9.73%), *Haemophilus* (9.67%), and *Veillonella* (7.27%). Similarly, the PI group was dominated by *Haemophilus* (13.41%), *Streptococcus* (13.34%), *Fusobacterium* (12.94%), *Rothia* (6.49%), and *Neisseria* (6.34%). Plots of longitudinal trajectories showed the pattern of shift in the most abundant genera by site group (Figure 2B, Supplementary Figure S1) and by individual site (Supplementary Figure S2). At 0 months, the top 3 abundant genera in the PI group were *Fusobacterium* (20.99%), *Streptococcus* (15.61%), and *Porphyromonas* (8.44%). In the HI group, the top 3 genera were *Streptococcus* (16.91%), *Fusobacterium* (11.39%), and *Neisseria* (10.06%). *Streptococcus* had a higher relative abundance in the HI group (16.91%) compared to the PI group (15.61%). In contrast, *Fusobacterium* was enriched in the PI group (20.99%) compared to the HI group (11.39%). At 3 months, the top 3 abundant genera in the PI group were *Haemophilus* (19.94%), *Streptococcus* (11.30%), and *Fusobacterium* (8.99%). In the HI group, the top 3 genera were *Fusobacterium* (13.17%), *Streptococcus* (12.30%), and *Haemophilus* (9.83%). *Haemophilus* emerged as the dominant genus in both groups, with higher levels in the PI group (19.94%) compared to the HI group (9.83%). *Fusobacterium* showed a higher relative abundance in the HI group (13.17%) compared to the PI group (8.99%). *Streptococcus* remained highly abundant in both groups. By 6 months, the top 3 abundant genera in the PI group were *Haemophilus* (17.70%), *Streptococcus* (13.10%), and *Fusobacterium* (8.84%). In the HI group, the top 3 genera were *Haemophilus* (17.36%), *Veillonella* (10.88%), and *Neisseria* (9.90%). *Haemophilus* maintained its dominance in both groups, with slightly higher abundance in the PI group (17.70%) compared to the HI group (17.36%). *Streptococcus* was consistently abundant, but higher in the PI group (13.10%) than in the HI group (8.82%).

### Alpha and beta diversity

**Fig. 3:**
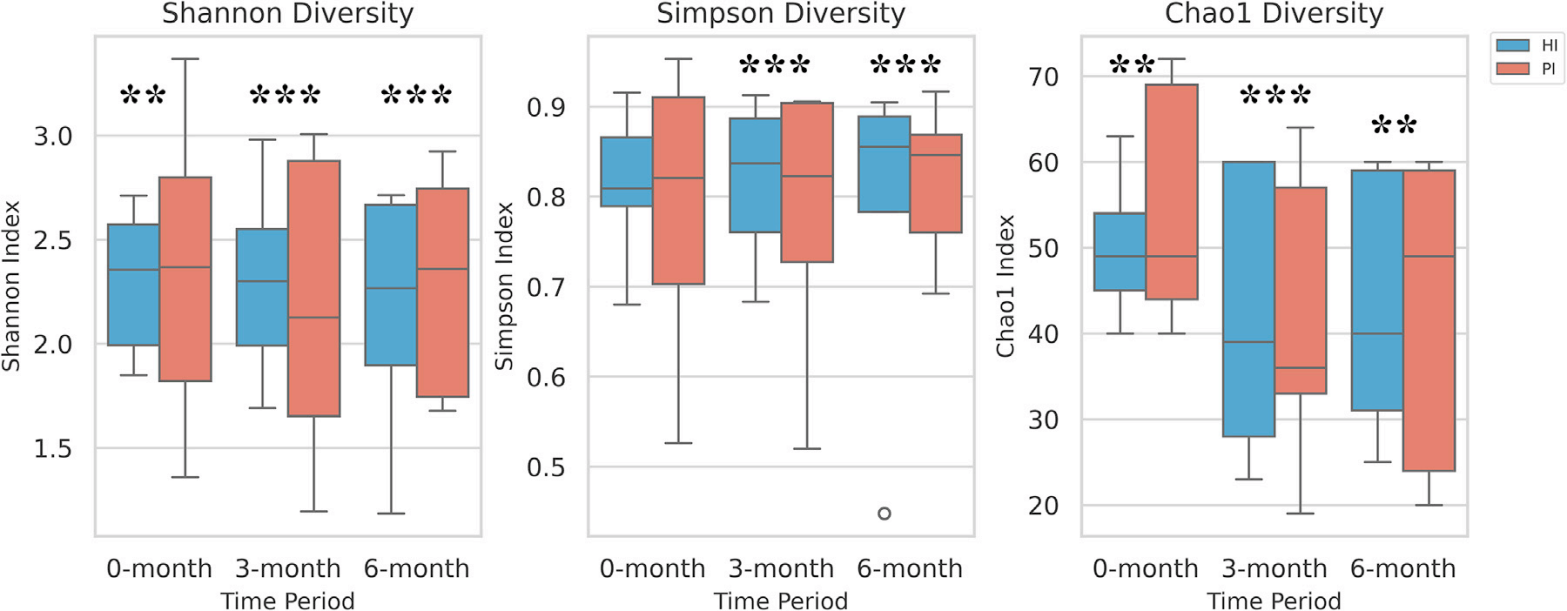
Bar plots depicting alpha diversity metrics in the 2 study groups. HI: Healthy Implants; PI: Peri-implantitis Implants; *P-value < .05, **P-value < .01, ***P-value < .001.

**Fig. 4:**
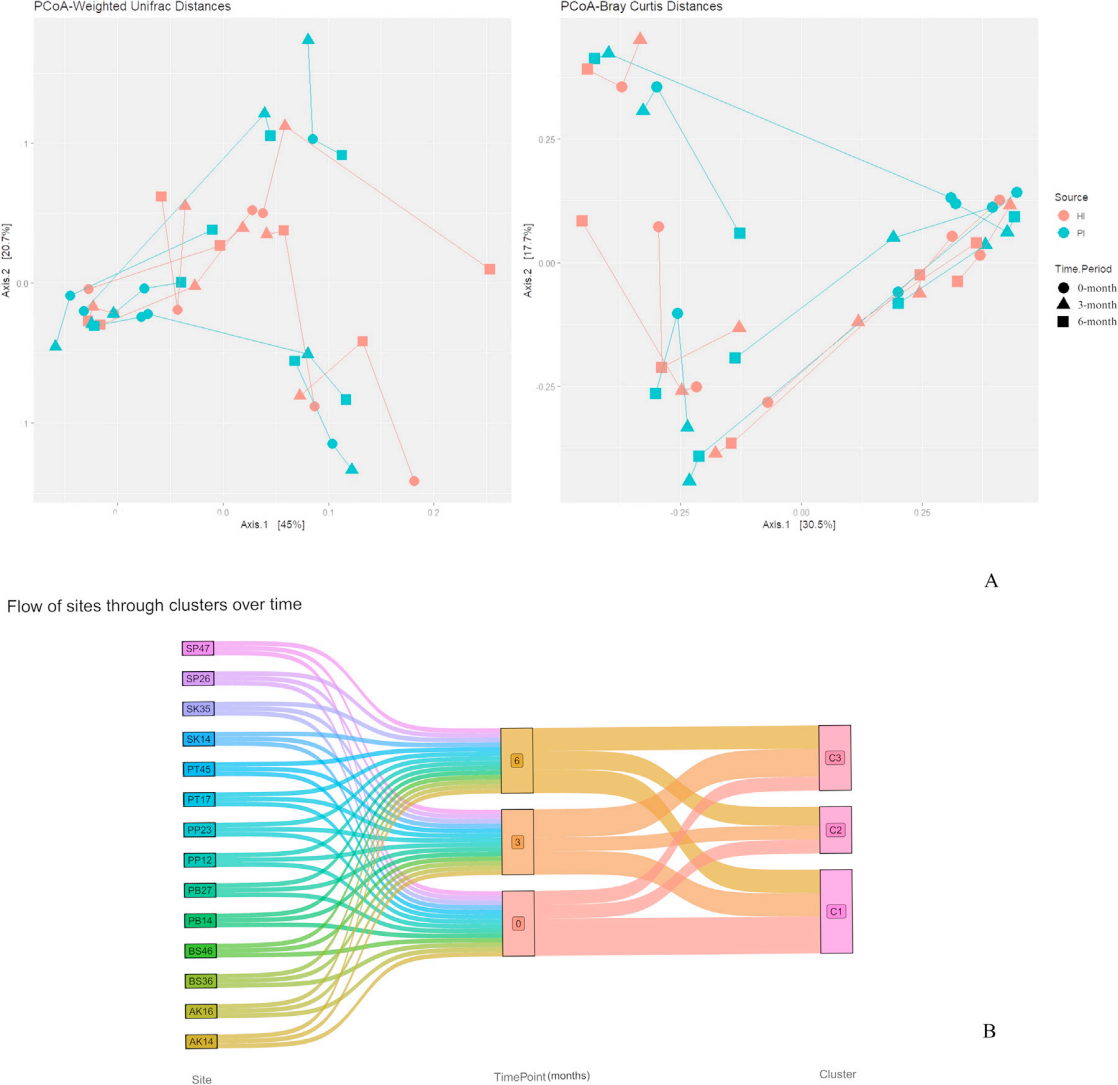
(A) Principal Coordinate Analysis (PCoA) using Weighted Unifrac and Bray-Curtis distance matrices. Samples are colored by HI/PI status and the shape represents the time point of sampling. Samples from the same subject are connected by lines. HI: Healthy Implants; PI: Peri-implantitis Implants. (B) Sankey plot depicting the transition of sites through the 3 microbiome-based clusters (C1, C2 and C3) over time. Cluster analysis was performed using the Bray-Curtis distance with the agglomerative Ward's hierarchical cluster algorithm and 3 clusters were selected to partition the samples.

Our analysis revealed statistically significant differences in alpha diversity indices between the HI and PI groups. Specifically, the Shannon index showed significant differences across all time points: 0-month (*P < .*01), 3-month (*P < .*001), and 6-month (*P < .*001). For the Simpson index, significant differences were observed at 3-month (*P < .*001) and 6-month (*P < .*001), while the Chao1 index demonstrated significant differences across all time points: 0-month (*P < .*01), 3-month (*P < .*001), and 6-month (*P < .*01) (Figure 3). Principal Coordinate Analysis revealed no distinct separation in overall microbiome composition between the PI and HI groups. Principal Coordinate Analysis revealed no distinct separation in overall microbiome composition between the PI and HI groups. There was no statistically significant clustering of PI and HI groups (R squared = 0.01, F = 0.59, *P = .*81) or that by time point (R squared = 0.04, F = 1.02, *P = .*39) by PERMANOVA Adonis (Figure 4A). However, significant differences were observed for inter-subject variability (R squared = 0.58, F = 2.98, *P = .*001) indicating that subject-level clustering was significant (Supplementary Figure S2). Cluster analysis using Bray-Curtis distances formed 3 distinct clusters (C1, C2, and,C3). The flow of sites through these clusters is shown in Figure 4B, and a corresponding dendrogram is displayed in Supplementary Figure S4. Additionally, the characteristics of each cluster are presented in Supplementary Table S13. The analysis showed that samples from the same site (*P = .*0006) and the same patient (*p* = 1.363e-05) were significantly associated with cluster membership, whereas the time of sample collection was not significantly associated (*P = .*699). Marker taxa analysis revealed that Cluster 1 was enriched in Fusobacteria, including *Fusobacterium nucleatum*. Cluster 2 was characterized by Gammaproteobacteria and Actinobacteria, including *Haemophilus parainfluenzae* and *Rothia dentocariosa*. Cluster 3 was marked by the enrichment of Burkholderiales (Supplementary Figure S5).

### Core microbiome analysis

**Fig. 5:**
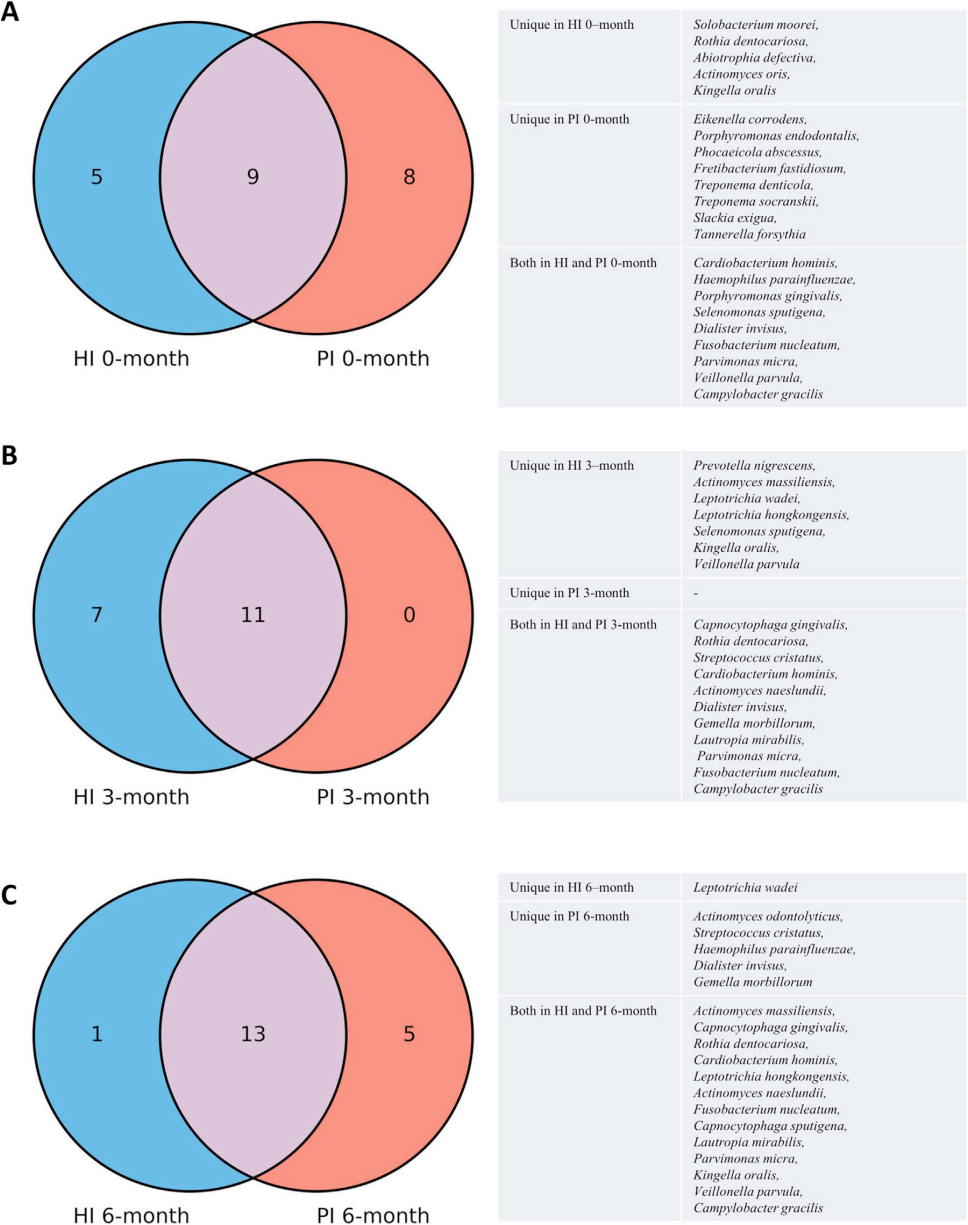
Venn diagram showing the union of core microbiomes (A) HI at 0 months and PI at 0 months, (B) HI at 3 months and PI at 3 months, (C) HI at 6 months and PI at 6 months. The table beside the diagram lists the species names included in the Venn diagram. HI: Healthy Implants; PI: Peri-implantitis Implants.

The core microbiome analysis identified distinct bacterial species consistently present at zero months in both the HI and PI groups. A total of 22 core species were identified at zero months, with 5 species unique to the HI group and 8 species specific to the PI group. At 3 months, the total number of core species was 18, including 7 species specific to the HI group, while no unique species were observed in the PI group. By 6 months, the total number of core species was 19**,** with 1 species uniquely found in the HI group and 5 species specific to the PI group (Figure 5A-C).

### Differentially abundant species

**Fig. 6:**
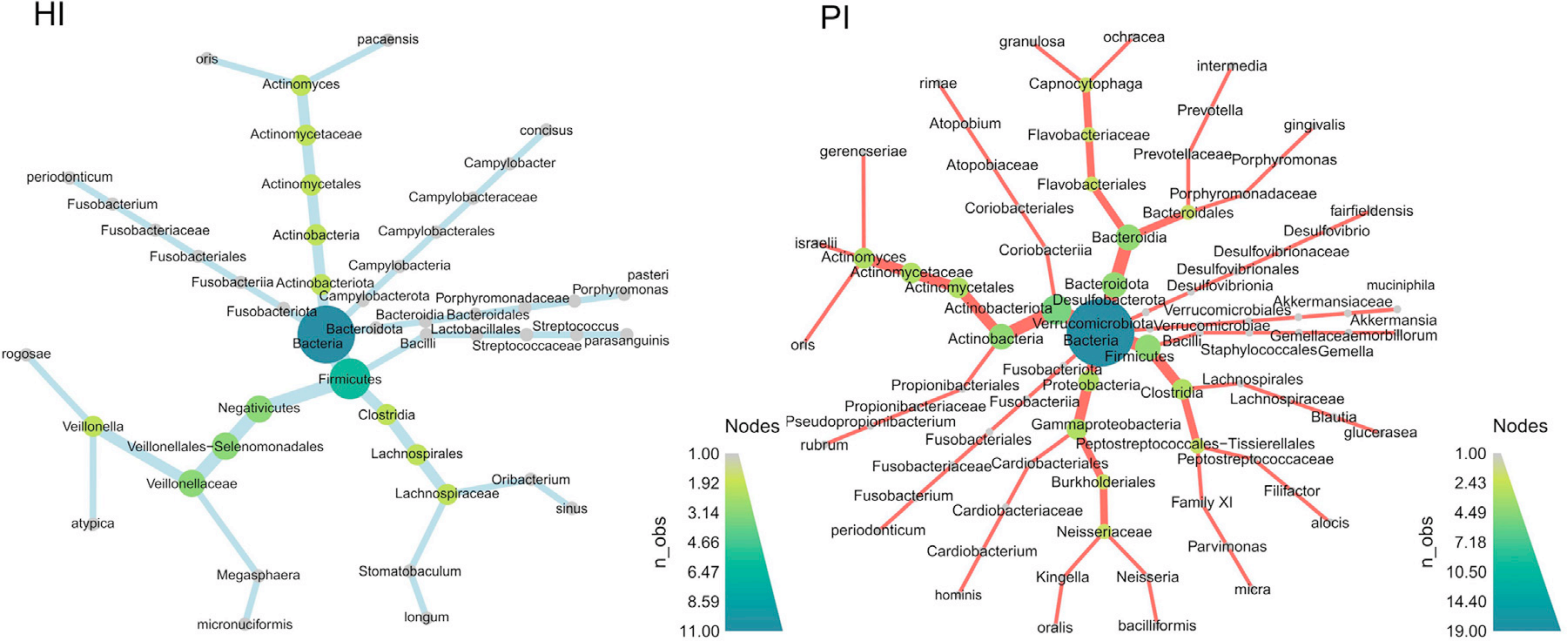
Heat trees depict the phylogenetic relationships of the differentially abundant species at all 3 time points. The heat tree on the left depicts species differentially enriched in the HI group and that on the right depicts species differentially enriched in the PI group. Node size corresponds to abundance. Detailed information is presented in Supplementary Tables S2-S4. HI: Healthy Implants; PI: Peri-implantitis Implants.

**Fig. 7:**
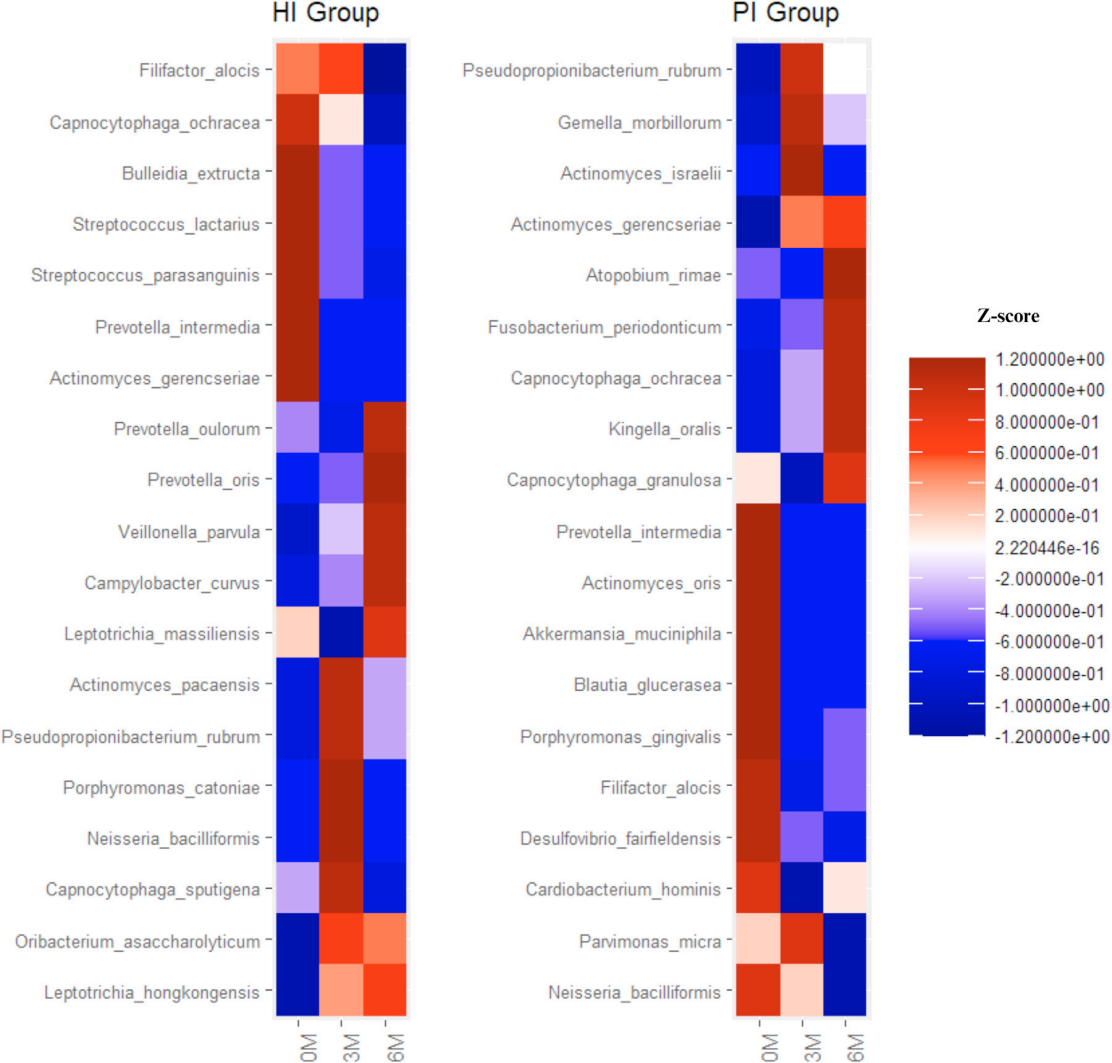
Heatmaps show the mean Z-transformed relative abundances of significantly differentially abundant taxa at 0-, 3-, and 6-month time points in the HI and PI groups, respectively. Detailed information is provided in Supplementary Tables S5–S10. HI: Healthy Implants; PI: Peri-implantitis Implants. 0M: 0-month: 3M: 3-month; 6M: 6-month.

Differentially abundant species between HI and PI groups at all 3 timepoints are depicted as heat trees (Figure 6; Supplementary Table S2-S4). In a comparison between the HI and PI groups at zero months, the PI group exhibited a significantly higher abundance of species, including *Neisseria bacilliformis, Porphyromonas gingivalis*, and *Prevotella oris*. In the HI group, 9 significantly higher abundances, including *Campylobacter concisus, Oribacterium sinus, Actinomyces gerencseriae, Fusobacterium periodonticum, Capnocytophaga ochracea, Stomatobaculum longum, Veillonella atypica, Streptococcus parasanguinis,* and *Porphyromonas pasteri*. At 3 months, the PI group demonstrated significantly higher abundances of *Phocaeicola abscessus, Actinomyces gerencseriae*, and *Anaeroglobus geminatus*. In the HI group, only *Actinomyces pacaensis* was significantly highly abundant. At 6 months, the PI group showed 5 significantly higher abundances of *Actinomyces gerencseriae, Streptococcus lactarius, Gemella bergeri, Capnocytophaga ochracea*, and *Johnsonella ignava*. In the HI group, 5 significantly higher species, including *Porphyromonas gingivalis, Megasphaera micronuciformis, Veillonella atypica, Actinomuces oris,* and *Veillonella rogosae*. Taxa that significantly differed in relative abundance within groups in HI and PI respectively are depicted as heat trees (Figure 7; Supplementary Table S5 -S10).

**Fig. 8:**
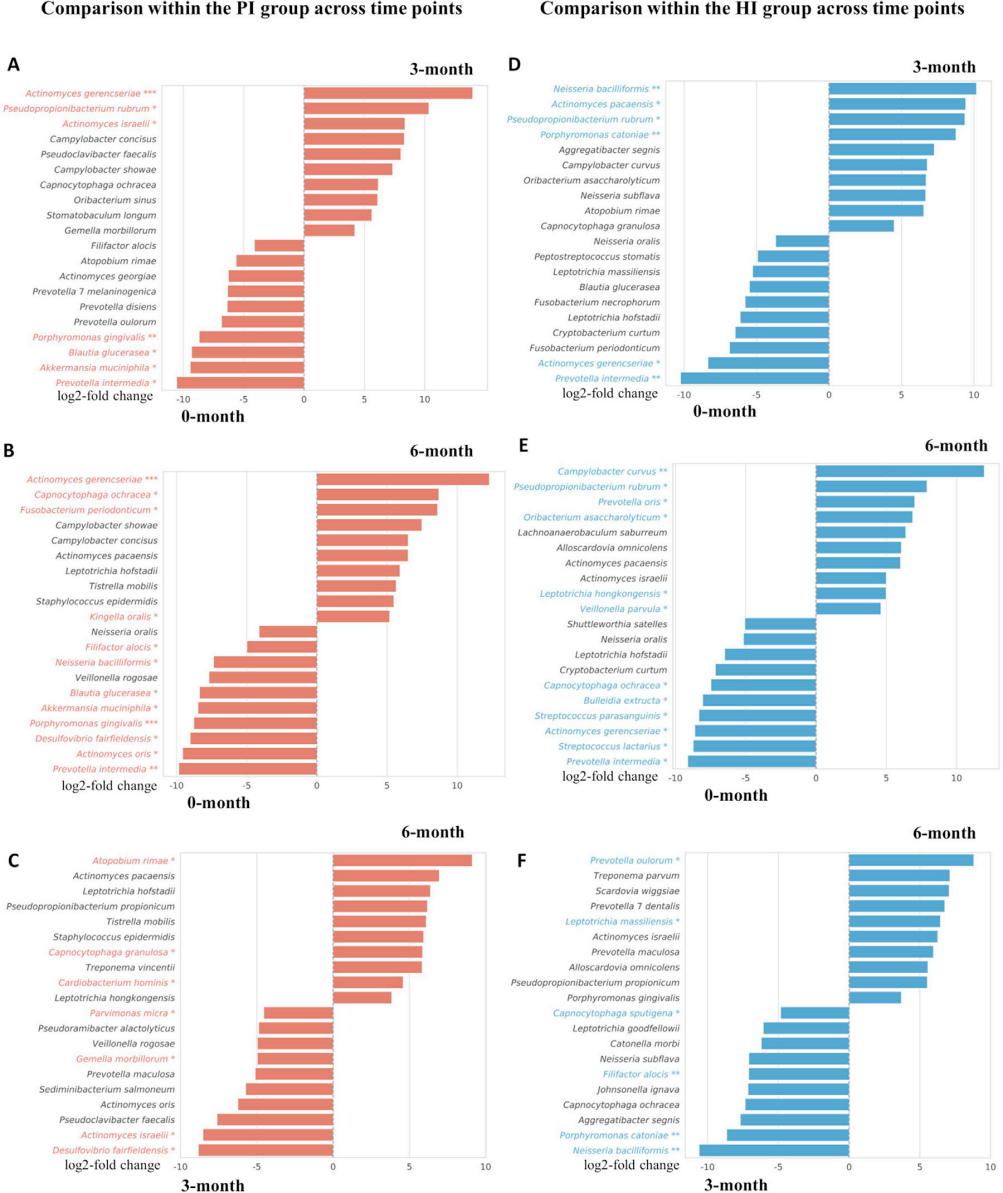
Differential abundance analysis of the top 10 species with positive and negative log2-fold change, (A) PI at 0 months versus PI at 3 months, (B) PI at 0 months versus PI at 6 months, (C) PI at 3 months versus PI at 6 months, (D) HI at 0 months versus HI at 3 months, (E) HI at 0 months versus HI at 6 months, (F) HI at 3 months versus HI at 6 months. The X axis represents a log2-fold change. Absolute higher log2-fold change value indicates a higher abundance (*p-value < .05, **p-value < .01, ***p-value < .001). HI: Healthy Implants; PI: Peri-implantitis Implants.

Comparison within the PI group across time points, at zero months, taxa significantly enriched in the PI group included *Porphyromonas gingivalis, Prevotella intermedia, Akkermansia muciniphila, Neisseria bacilliformis, Filifactor alocis, Desulfovibrio fairfieldensis*, and *Blautia glucerasea*. At 3 months post-treatment, the PI group demonstrated enrichment of *Actinomyces gerencseriae, Pseudopropionibacterium rubrum*, and *Actinomyces israelii*. By 6 months, the PI group showed significantly higher abundances of *Actinomyces gerencseriae, Kingella oralis, Capnocytophaga ochracea*, and *Fusobacterium periodonticum* (Figure 8A-B). When comparing 3 months to 6 months post-treatment, the PI group exhibited higher abundances of *Desulfovibrio fairfieldensis, Actinomyces israelii, Gemella morbillorum*, and *Parvimonas micra* at 3 months, while species such as *Atopobium rimae, Capnocytophaga granulosa*, and *Cardiobacterium hominis* were significantly enriched at 6 months (Figure 8C).

Comparison within the HI group across time points at 3 months, *Neisseria bacilliformis, Actinomyces pacaensis, Pseudopropionibacterium rubrum,* and *Porphyromonas catoniae* were significantly enriched, while zero months showed higher abundances of *Actinomyces gerencseriae* and *Prevotella intermedia* (Figure 8D). At 6 months, taxa such as *Campylobacter curvus, Pseudopropionibacterium rubrum*, and *Veillonella parvula* were significantly enriched (Figure 8E). Comparing 3 months to 6 months, *Capnocytophaga sputigena, Filifactor alocis, Porphyromas catoniae,* and *Neisseria bacilliformis* were more abundant at 3 months (Figure 8F). Additionally, the details of the differential analysis at each time point, highlighting the 10 highest and 10 lowest abundant species, are provided in Supplementary Table S2-S10.

**Fig. 9:**
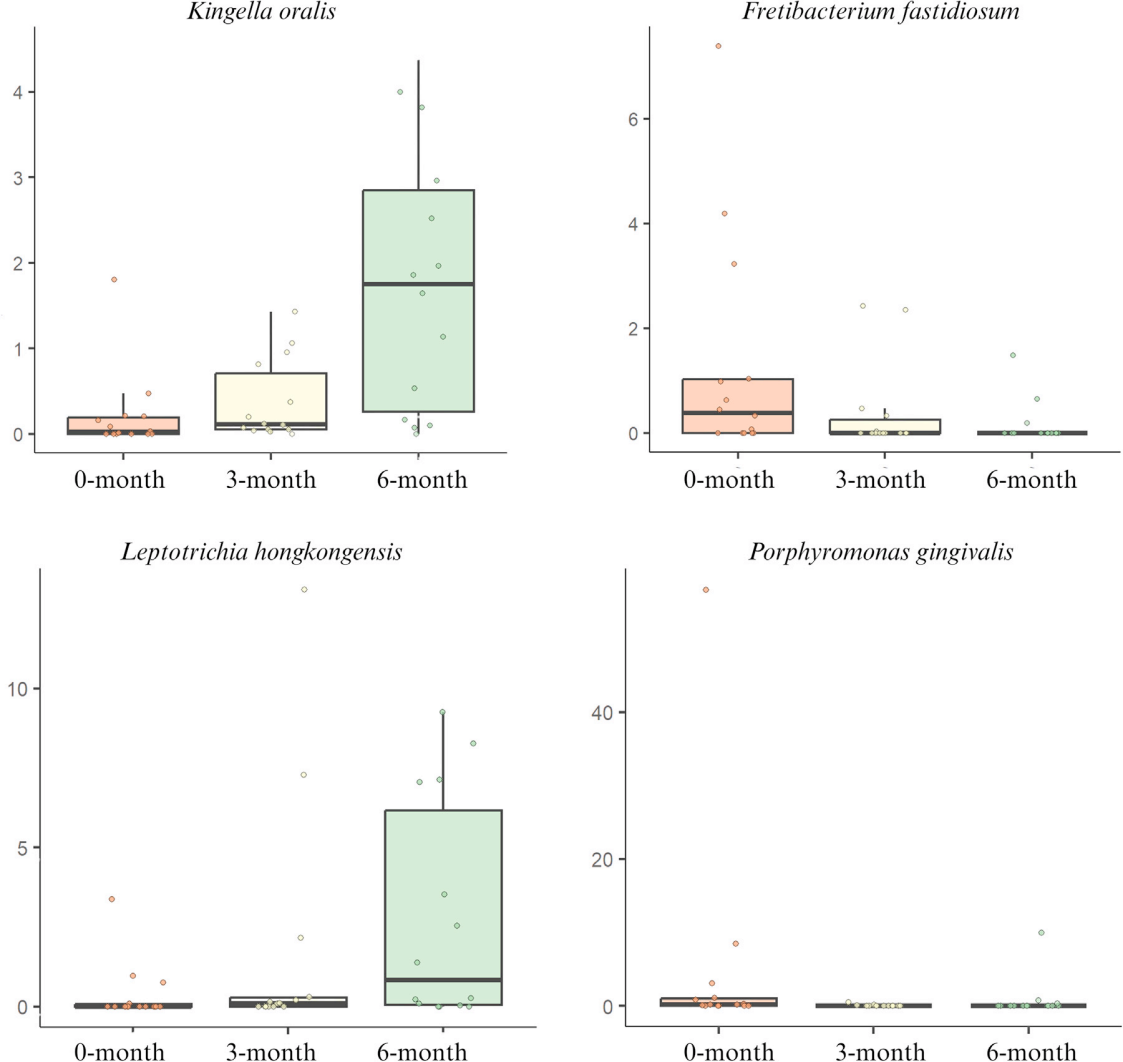
Boxplots show the relative abundances of 4 significantly differentially abundant species across the 3 time points, after controlling for inter-subject variation and peri-implant disease status using MaAsLin multivariate analysis.

MaAsLin multivariate testing, which adjusted for inter-subject variability and disease status, showed that *Kingella oralis* (*P < .*001) and *Leptotrichia hongkongensis* (*P < .*01) were significantly enriched in abundance at 6 months. In contrast, *Porphyromonas gingivalis* (*P < .*01) was significantly depleted at 3 months, and *Fretibacterium fastidiosum* (*P < .*01) was significantly depleted at 6 months post-treatment. These significant species-level changes are illustrated in Figure 9.

### Pathway analysis

**Fig. 10:**
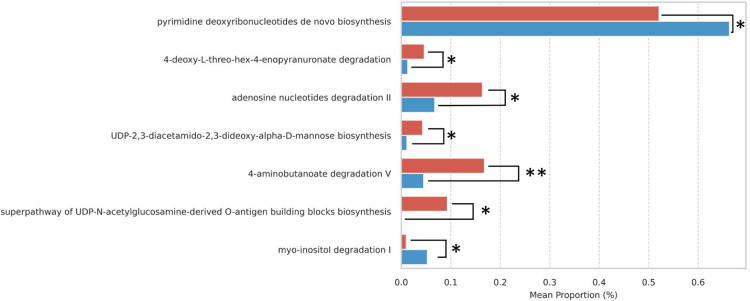
The percent mean proportion of selected significant pathways from the comparison between HI and PI at 0 months. HI: Healthy Implants; PI: Peri-implantitis Implants. *p-value < .05, **p-value < .01, ***p-value < .001.

A total of 287 pathways were predicted from our results, highlighting key differences between the PI and HI groups. Using a significance threshold of *p*-value < .05, several pathways were significantly enriched in PI compared to HI at zero months. These pathways include 4-deoxy-L-threo-hex-4-enopyranuronate degradation, adenosine nucleotide degradation II, UDP-2,3-diacetamido-2,3-dideoxy-α-D-mannuronate biosynthesis, 4-aminobutanoate degradation V, and the superpathway of UDP-N-acetylglucosamine-derived O-antigen building blocks biosynthesis. In contrast, pyrimidine deoxyribonucleotide de novo biosynthesis and myo-inositol degradation I was significantly enriched in HI compared to PI at zero months (Figure 10; Supplementary Table S11). However, no statistically significant differences were observed between the HI and PI groups at 3 months and 6 months post-treatment.

## Discussion

This study prospectively investigated microbial shifts at implant sites undergoing peri-implantitis treatment, revealing a clear association between clinical improvement and microbiome changes. At the community level, inter-subject variation was found to be the major driver of peri-implant microbiome composition across health states and time. A key finding is the significant reduction of both Fusobacteriota and Synergistetes phyla following treatment, previously been implicated in peri-implantitis^27^ and periodontal disease,^28^ respectively. This decrease highlights the effectiveness of the intervention in targeting key pathogens. Complementing this observation is the parallel reduction in Synergistetes, another phylum linked to periodontal disease, further underscoring the positive impact of therapy on the overall microbial ecology.

In this study, we evaluated the temporal dynamics of microbial communities following periodontal surgery using a within-subject, paired-site design. Each participant contributed both a diseased/treated site and a healthier/control site, which minimized inter-individual variability and allowed a focus on localized microbial shifts. This paired-site approach enabled the assessment of microbial changes relative to baseline for each individual. An increase in genera such as Haemophilus (Supplementary Figure S3).

Owing to the small number of subjects and the within-subject comparative design, we did not analyze the influence of subject-level parameters such as diabetes, history of periodontitis, age, and gender on microbiome composition. However, the robust effect of subject-level differences in microbiome composition was evident from the cluster and ANOSIM analyses (see Supplementary Table S13 and Figure S2). This finding warrants further investigation into the subject-specific and functional aspects of the microbiome in peri-implantitis pathology and treatment recovery.

Interestingly, we observed a dynamic interplay between pathogenic and beneficial bacteria throughout the treatment course. At baseline, the peri-implantitis group exhibited a core microbiome dominated by "red complex" members like *Tannerella forsythia* and *Treponema denticola*, known drivers of peri-implantitis progression.^29^ *Tannerella forsythia* is known to play a critical role in the disease's progression.^30^ By 6 months, the emergence of *Streptococcus cristatus* may suggest a shift towards a healthier microbial profile. This is particularly noteworthy given *Streptococcus cristatus*'s documented antagonistic activity against periodontal pathogens, including its suppression of *Porphyromonas gingivalis* virulence factors and modulation of *Fusobacterium nucleatum*-induced inflammation.^31^^,^^32^ This potential synergistic interaction between *Streptococcus cristatus* and treatment efficacy warrants further investigation.

We performed a cluster analysis based on microbiome profiles and identified 3 clusters of samples that were significantly associated with both site and subject. Cluster 1, marked by Fusobacteria enrichment, showed the highest proportion of pretreatment sites and included 4 sites from 3 patients that did not transition to other clusters post-treatment. However, at 3 and/or 6 months, 1 site transitioned to Cluster 2 and 3 sites transitioned to Cluster 3. Cluster 2, characterized by *Haemophilus parainfluenzae* and *Rothia dentocariosa,* included 2 sites that did not transition across time, while 1 baseline site transitioned to Cluster 3 post-treatment. Cluster 3, marked by Burkholderiales, included 1 site that did not transition from any other cluster. This analysis corroborated the findings of a robust effect size of inter-patient and inter-site variation on the peri-implant site microbiome and Fusobacterium depletion with treatment.

The top 10 most abundant genera showed differences between peri-implantitis (PI) and healthy implant (HI) sites (Figure 2B, Supplementary Figure S1), which are relevant to peri-implant disease pathogenesis, immune modulation, and biofilm dynamics.

The genus *Fusobacterium*, associated with periodontal and peri-implant inflammation,^27^ showed a notable decrease in PI sites at 3 months post-treatment, while remaining relatively stable in HI sites. This trend aligns with prior studies reporting a decrease in *Fusobacterium* abundance in PI sites following treatment.^30^ The observed reduction in PI sites suggests a positive therapeutic response and a potential shift toward a less pathogenic microbial profile. However, the persistence of Fusobacterium abundance at 6 months in peri-implant sites suggests it may serve as a microbial risk indicator for disease recurrence following surgical intervention. This finding is consistent with previous systematic reviews indicating that *Fusobacterium nucleatum* often remains stable or is only marginally reduced even after therapeutic intervention.^30^ In addition, *Fusobacterium nucleatum* can directly invade epithelial and endothelial host cells,^33^ which may allow it to evade immune responses and resist therapeutic interventions targeting oral diseases, systemic inflammatory conditions, and even neoplasms located beyond the oral cavity.^34^^,^^35^

The genus *Haemophilus* is typically regarded as a commensal organism in healthy oral biofilms^36^ and showed an increase at 3 months in both PI and HI sites. Previous research demonstrated that *Haemophilus parainfluenzae* possesses a core set of essential genes for survival and biofilm growth, including conditionally essential genes that support its fitness in both aerobic and anaerobic environments.^37^ This genetic versatility may allow it to persist within the biofilm core and evade elimination during standard mechanical or antimicrobial therapy.

*Veillonella*, an obligate anaerobe, increased at 3 and 6 months in both HI and PI sites. It helps maintain oral homeostasis by fermenting lactate into short-chain fatty acids like acetate and propionate. *Veillonella dispar* adapts its metabolism under nutrient-limited conditions, boosting SCFA production,^38^ which supports immune modulation, tissue repair, and microbiome stability.

The genus *Capnocytophaga*, comprising Gram-negative, facultatively anaerobic rods and constituents of the normal oral microbiota, exhibited a marked increase in both HI and PI sites 3 months post-treatment. Capnocytophaga species are strongly associated with periodontal health^38^ and typically demonstrate lower abundance in peri-implantitis.^39^ Consequently, their post-treatment proliferation in PI sites may signify early recolonization or compensatory microbial shifts during community restructuring, potentially fostering the re-establishment of a more balanced, health-compatible biofilm.

The genus *Leptotrichia*, a component of the normal oral microbiota, also increased in both HI and PI sites 3 months post-treatment. While previously identified in peri-implantitisn^40^ and implicated in complex host-microbe interactions during the health-to-disease transition, *Leptotrichia* can induce pro-inflammatory cytokine transcription in epithelial cells, thereby modulating epithelial immunity.^41^ The moderate post-treatment increase in *Leptotrichia* observed in our study may thus represent recolonization dynamics rather than a definitive pathogenic shift.

*Neisseria,* a common constituent of human mucosal microbiota, is typically more prevalent in healthy peri-implant sites, suggesting a role in microbial homeostasis.^39^ In this study, Neisseria levels remained stable in HI sites and increased in PI sites 3 months post-treatment. The sustained relative abundance of this health-associated genus in HI sites concurs with previous reports linking Neisseria to peri-implant health.^42^ Its modest post-treatment increase in PI sites may indicate initial microbial community recovery towards stability.

The genus *Rothia,* a typically commensal but potentially opportunistic bacterium, decreased in HI sites while increasing in PI sites 3 months post-treatment. Its reduction in HI sites may suggest niche competition or ecological succession during healing. Conversely, its post-treatment increase in PI sites could indicate compensatory colonization during microbial reassembly, aligning with previous findings associating *Rothia* with peri-implant health and microbial stabilization during recovery.^43^

The genus *Streptococcus*, a common peri-implant colonizer involved in biofilm formation and potential disease progression,^44^ decreased in both HI and PI sites 3 months post-treatment. This reduction, especially in PI sites post-intervention, may signify a transition to a health-compatible microbial community, considering reported associations between specific Streptococcus species and peri-implantitis.^44^

*Actinomyces* abundance increased in both the PI and HI sites over 3 months. This aligns with prior studies suggesting its protective role against pathogenic colonization, fostering a balanced, health-conducive microbial ecosystem.^42^ The increase, particularly in PI sites, may therefore indicate a shift towards health-associated microbial communities during healing.

The genus *Parvimonas*, implicated in peri-implantitis progression,^45^ increased in both HI and PI sites 3 months post-treatment. This contrasts with reports of its typical decline following therapy at this timepoint,^46^ potentially indicating incomplete inflammation resolution or early site recolonization. However, longitudinal analysis revealed a marked decline in PI sites by 6 months, aligning with findings of decreased Parvimonas micra levels after mechanical and antiseptic treatment of peri-implant lesions.^47^ This delayed reduction suggests Parvimonas may temporarily occupy an ecological niche during early healing, eventually being suppressed as immune responses stabilize and a more health-associated microbiome re-establishes. Therefore, its initial persistence likely reflects transient dysbiosis rather than sustained pathogenicity.

Differential abundance analysis revealed additional insights into these microbial dynamics. Initially, the presence of established periodontal pathogens like *Porphyromonas gingivalis* and *Prevotella oris* corroborated with the disease state.^11^^,^^48^ *Porphyromonas gingivalis* is known to be involved in triggering inflammatory processes and associated with peri-implantitis.^39^^,^^49^ However, post-treatment, we observed an increase in the relative abundance of beneficial species such as *Actinomyces gerencseriae*, associated with oral health,^42^ and a concurrent absence of peri-implantitis-associated pathogens. This transition was further reinforced at 6 months with the increased abundance of *Streptococcus lactarius* and *Gemella bergeri*, both indicative of a healthy oral microbiome.^42^^,^^50^^,^^51^ This sequential shift in microbial composition provides compelling evidence for the treatment's effectiveness in promoting a healthy oral environment. Importantly, the increase in *Akkermansia muciniphila* 3 months post-treatment may be clinically relevant, given its reported anti-inflammatory properties and potential role in inhibiting periodontitis associated with *Fusobacterium nucleatum*.^52^^,^^53^ This suggests a potential synergistic effect between the treatment and the emergence of this beneficial species. This suggests a potential synergistic effect between the treatment and the emergence of this beneficial species. At the same time, using multivariate testing to control for the effects of inter-subject variation and implant health status, 2 species *Kingella oralis* and *Leptotrichia hongkongensis* were found to significantly increase at the 6-month time point independent of disease status. *Kingella oralis* has been noted in periodontitis and peri-implantitis,^54^ whereas *Leptotrichia hongkongenis* has been noted in peri-implant sites.^55^ The plasticity of the peri-implant microbial community in health and disease and its implications warrant further investigation.

Our study also revealed significant differences in predicted microbial functional pathways between the PI and HI groups at baseline, providing potential mechanistic insights into microbial activities contributing to peri-implant disease pathogenesis or resolution. Notably, the pyrimidine deoxyribonucleotide de novo biosynthesis pathway, crucial for DNA replication and implicated in virulence factor production, was significantly more abundant in the HI group than in the PI group. This contrasts with earlier findings associating higher activity of this pathway with bacterial pathogenesis.^56^ The observed lower abundance in the PI group may reflect microbial stress or suppressed proliferation under inflammatory conditions.

The 4-deoxy-L-threo-hex-4-enopyranuronate degradation pathway, involved in short-chain fatty acid (SCFA) biosynthesis,^57^ was also differentially abundant, being more enriched in the PI group than in the HI group at baseline. Prior studies have demonstrated that elevated levels of SCFAs (e.g., butyric, isobutyric, and isovaleric acids) are associated with cytotoxicity and tissue damage linked to peri-implant disease.^58^ Thus, the enrichment of this pathway in the PI group at baseline aligns with reports linking SCFA accumulation to peri-implant inflammation. Conversely, there was a concurrent enrichment of the 4-aminobutanoate degradation pathway in the HI group. This pathway is commonly associated with protective, butyrate-producing microbiota, supporting a metabolic shift from a disease-associated to a health-associated microbial profile.^59^ Furthermore, pathways related to lipopolysaccharide (LPS) biosynthesis, including the superpathway of UDP-N-acetylglucosamine-derived O-antigen building block biosynthesis, was upregulated in the PI group compared to the HI group.^60^ LPS, a major component of the Gram-negative bacterial outer membrane, comprises lipid A, the core oligosaccharide, and the O-antigen.^61^ The dysregulation of LPS-associated pathways in the PI group may indicate heightened inflammatory activity, potentially mediated via toll-like receptor 4 (TLR4) signaling, consistent with evidence linking microbiota-derived LPS to inflammation.^62^

The myo-inositol degradation I pathway, implicated in biofilm formation, was enriched in the HI group. This pathway catabolizes myo-inositol, and its metabolism reportedly enhances biofilm-associated gene expression, supporting microbial survival, cohesion, and structured community establishment.^63^ Its enrichment in HI suggests health-associated consortia utilize inositol-derived substrates for stable biofilm architecture. Conversely, its underrepresentation in the PI group may reflect a disrupted, dysbiotic microbial structure less reliant on this cooperative metabolism, consistent with inflammatory peri-implant disease. Finally, the adenosine nucleotide degradation II pathway, a purine degradation pathway,^64^ was more enriched in the PI group. It catalyzes adenosine breakdown into metabolites (inosine, hypoxanthine, and uric acid) known to impact immune cell activation, oxidative stress, and tissue inflammation.^65^ Its elevated abundance in PI sites suggests a microbial metabolic shift potentially modulating host immune responses in the peri-implant niche.

Several species associated with the post-treatment healthy status could be potential probiotic species, warranting deeper investigation. Thus, the observed shifts in the microbiome composition following peri-implantitis treatment have significant clinical implications. The reduction of pathogenic bacteria, coupled with the emergence of beneficial commensals like *Streptococcus cristatus* and *Actinomyces gerencseriae*, suggests the potential for targeted therapies aimed at modulating the peri-implant microbiome. Specifically, future research could explore prebiotic or probiotic approaches to promote the growth of beneficial bacteria and inhibit the colonization of pathogens, potentially enhancing treatment outcomes and long-term implant success. The identification of *Akkermansia muciniphila* as a potential player in post-treatment health warrants further investigation into its role and potential therapeutic applications. Understanding the complex interplay between the microbiome and host response in peri-implantitis could pave the way for personalized treatment strategies that optimize microbial balance and promote sustained peri-implant health. Importantly, the subject level clustering of longitudinal peri-implant microbiome composition in both health and disease suggests a necessity for a whole-mouth treatment approach for risk mitigation and longitudinal studies correlating treatment responses to microbiome and host response features. Of note, an absence of BOP in response to treatment was noted in a subgroup of implants with PI, but owing to the limited sample size subject level stable or plastic microbiome features predictive of such response were not determined.

Several limitations of this study warrant acknowledgment. The limited sample size constrained the statistical power of our analyses and may restrict the generalizability of the findings to broader patient populations. Although the within-subject, paired-site design enhanced internal validity by minimizing inter-individual variability, it may not have entirely mitigated the influence of all potential confounding factors. Variables such as systemic health conditions (e.g., diabetes), prior surgical interventions, and oral hygiene practices were not controlled for in the current study. Furthermore, the reliance on 16S rRNA gene sequencing may not have provided a complete characterization of the oral microbiome, particularly concerning functional pathways, as these were inferred using predictive bioinformatics tools. Future research should aim to incorporate larger sample sizes to achieve greater statistical power and implement more rigorous control over sample conditions for a more comprehensive analysis. The application of whole-genome shotgun sequencing would also enable a more in-depth and comprehensive examination. Moreover, the integration of multi-omics data, including metagenomics, metatranscriptomics, and host-response profiles, will be crucial for elucidating more precise mechanistic insights into the microbial dynamics associated with peri-implant health, disease, and healing.

## Conclusion

This study provides valuable insights into the dynamic shifts in microbial communities in peri-implantitis sites following treatment over a 6-month period. The significant reduction in pathogenic bacteria such as *Porphyromonas gingivalis* and *Prevotella intermedia* highlights the treatment's efficacy in reducing disease-associated species. Additionally, core microbiome analysis revealed further distinctions between the PI and HI groups. The PI group was primarily populated with periodontal pathogens such as *Tannerella forsythia* and *Treponema denticola*.

## Conflicts of interest

The authors declare that there is no conflict of interest regarding the publication of this article.

## Appendix Supplementary materials

Image, application 1

## Footnotes

Supplementary material associated with this article can be found in the online version at doi:10.1016/j.identj.2025.100951.
