# Supplementary material for: Microbiome Shifts in Peri-Implantitis: Longitudinal Characterization of Dysbiosis and Resolution
Source: Int Dent J. 2025 Aug 13;75(5):100951. doi: 10.1016/j.identj.2025.100951 (PMC12361765; doi:10.1016/j.identj.2025.100951)
Supplement: Supplementary file 1 [file mmc1.docx]

**Supplementary Data**

**Microbiome Shifts in Peri-Implantitis: Longitudinal Characterization of Dysbiosis and Resolution**

**
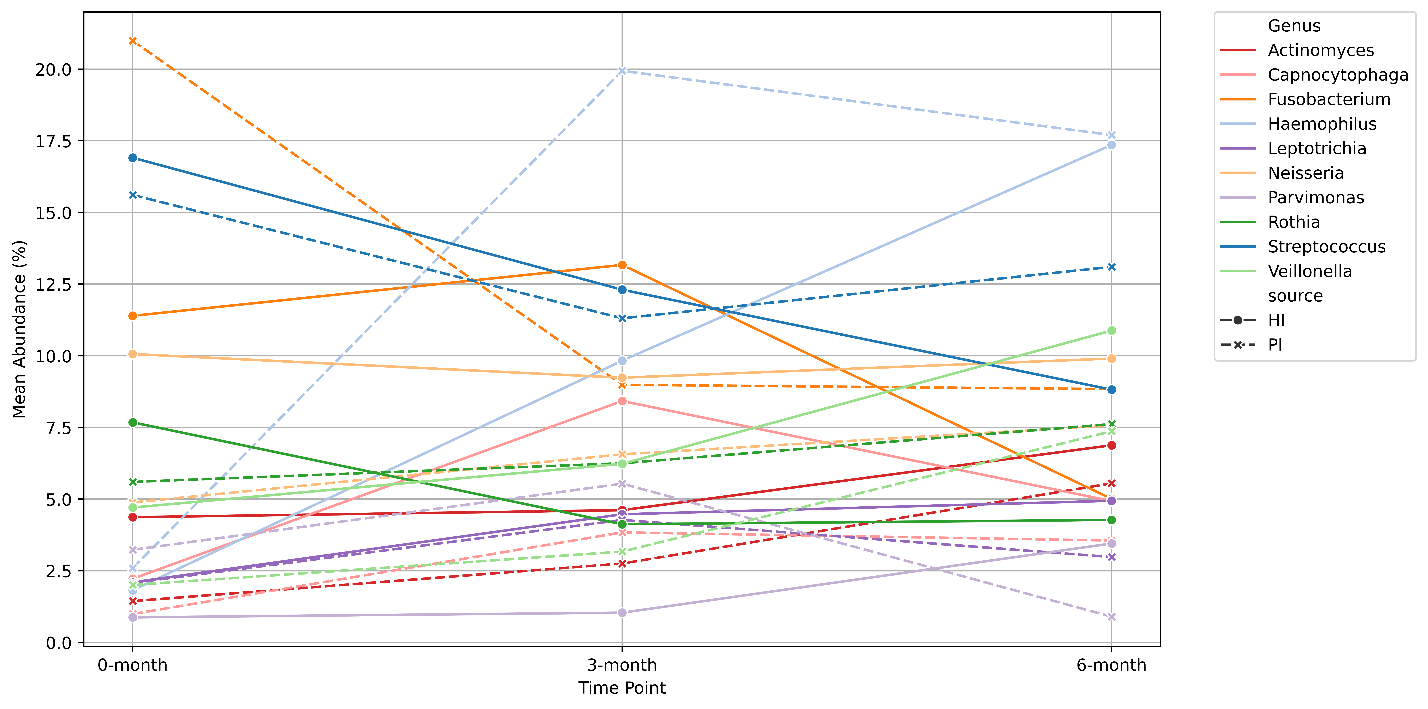
**

**Supplementary Figure S1** Mean relative abundance of microbial taxa at the genus level over time. The temporal changes in the mean relative abundance (%) of top microbial taxa across three clinical time points: 0-month (baseline), 3-month, and 6-month. HI: Healthy Implants; PI: Peri-implantitis Implants.

**
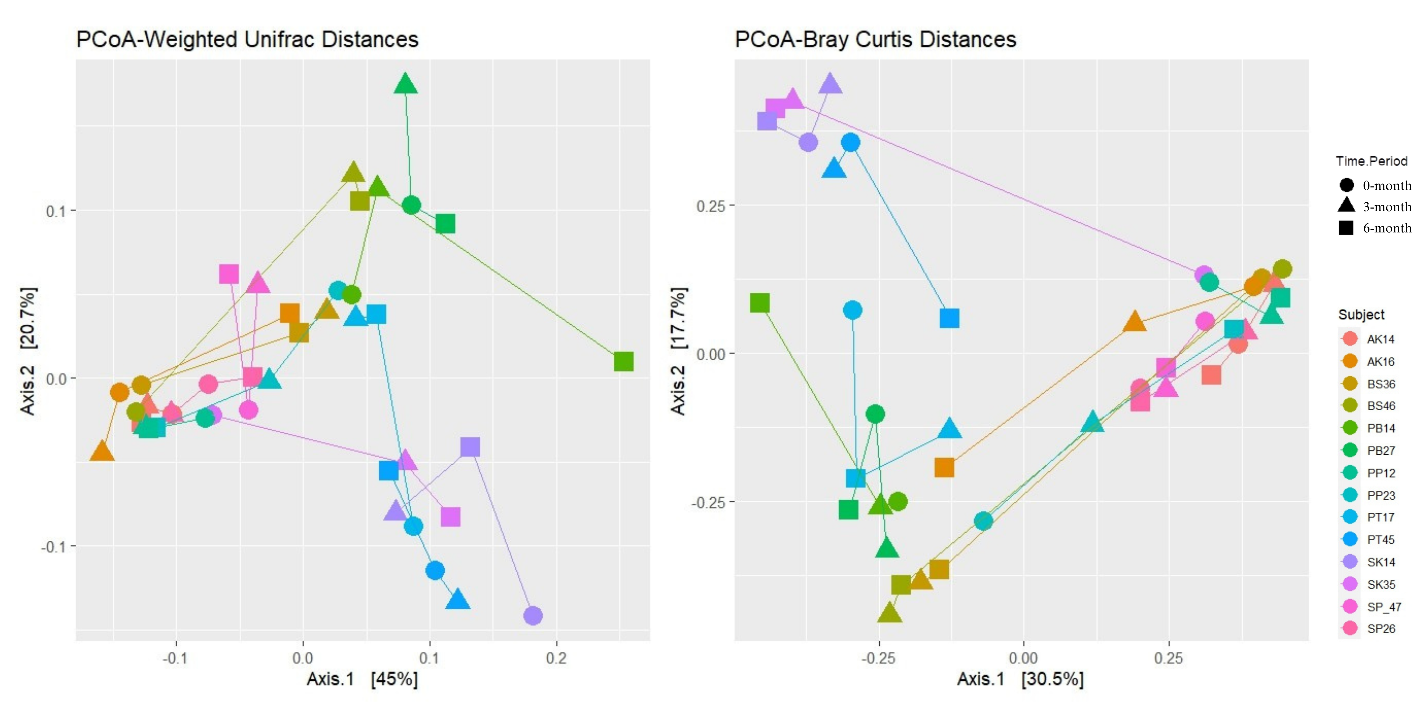
**

**Supplementary Figure S2** Principal coordinate analysis of Bray Curtis and Weighted Unifrac distances, with samples colored by Subject ID. Lines connect samples from the same subject and point shapes correspond to disease status of implants. Notable, AK and PT included diabetic which showed the similarly cluster to others participant.


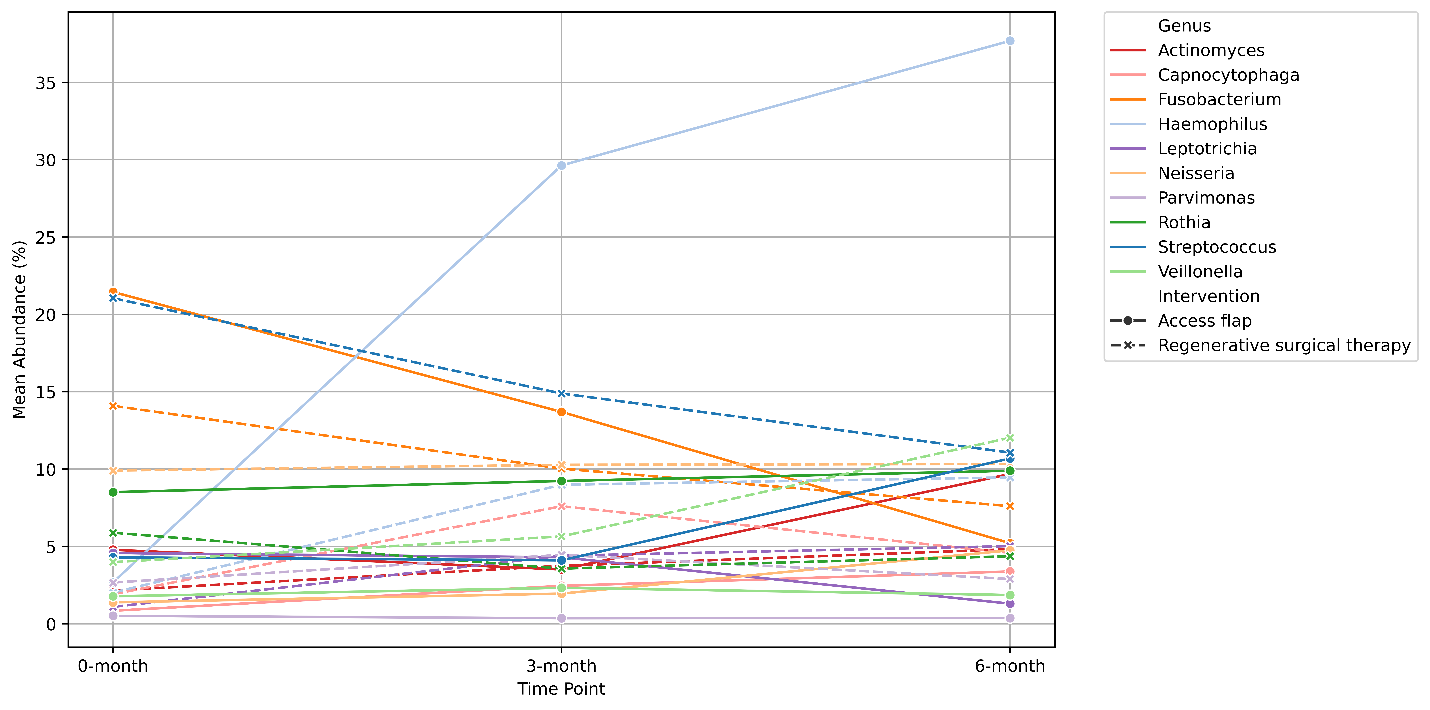


**Supplementary Figure S3** Mean relative abundance of microbial taxa at the genus level over time. The temporal changes in the mean relative abundance (%) of the top bacterial genera across three clinical time points: 0-month (baseline), 3-month, and 6-month. Solid lines represent the Access flap group, and dashed lines represent the Regenerative surgical therapy group.

**
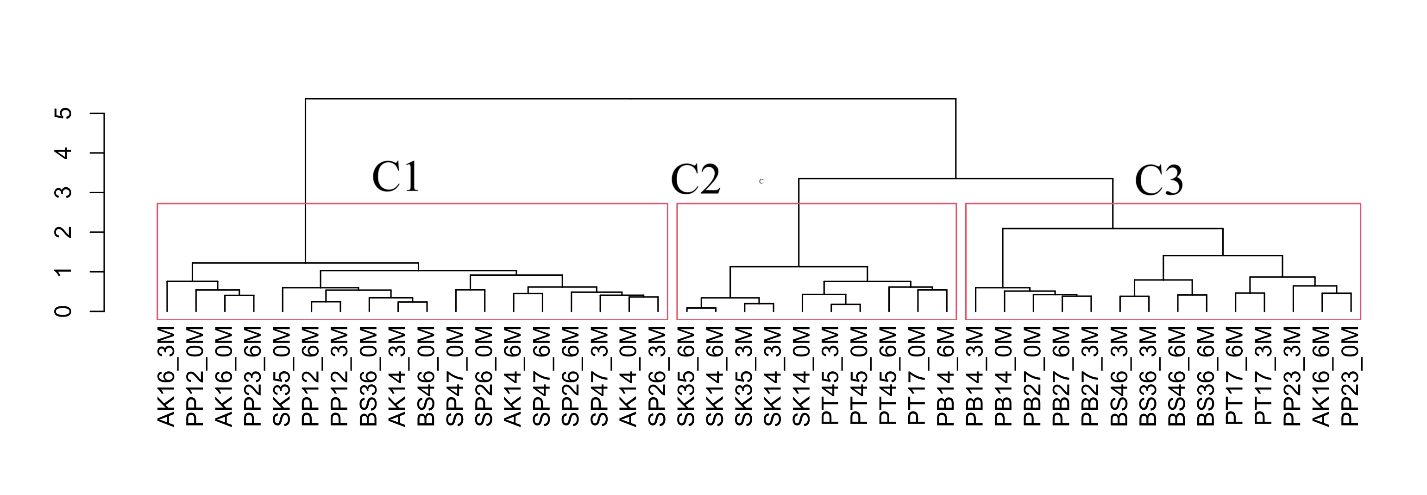
**

**Supplementary Figure S4** A dendrogram depicting the clustering of samples by agglomerative Ward's hierarchical cluster algorithm. Rectangles enclose the 3 clusters partitioning the samples.


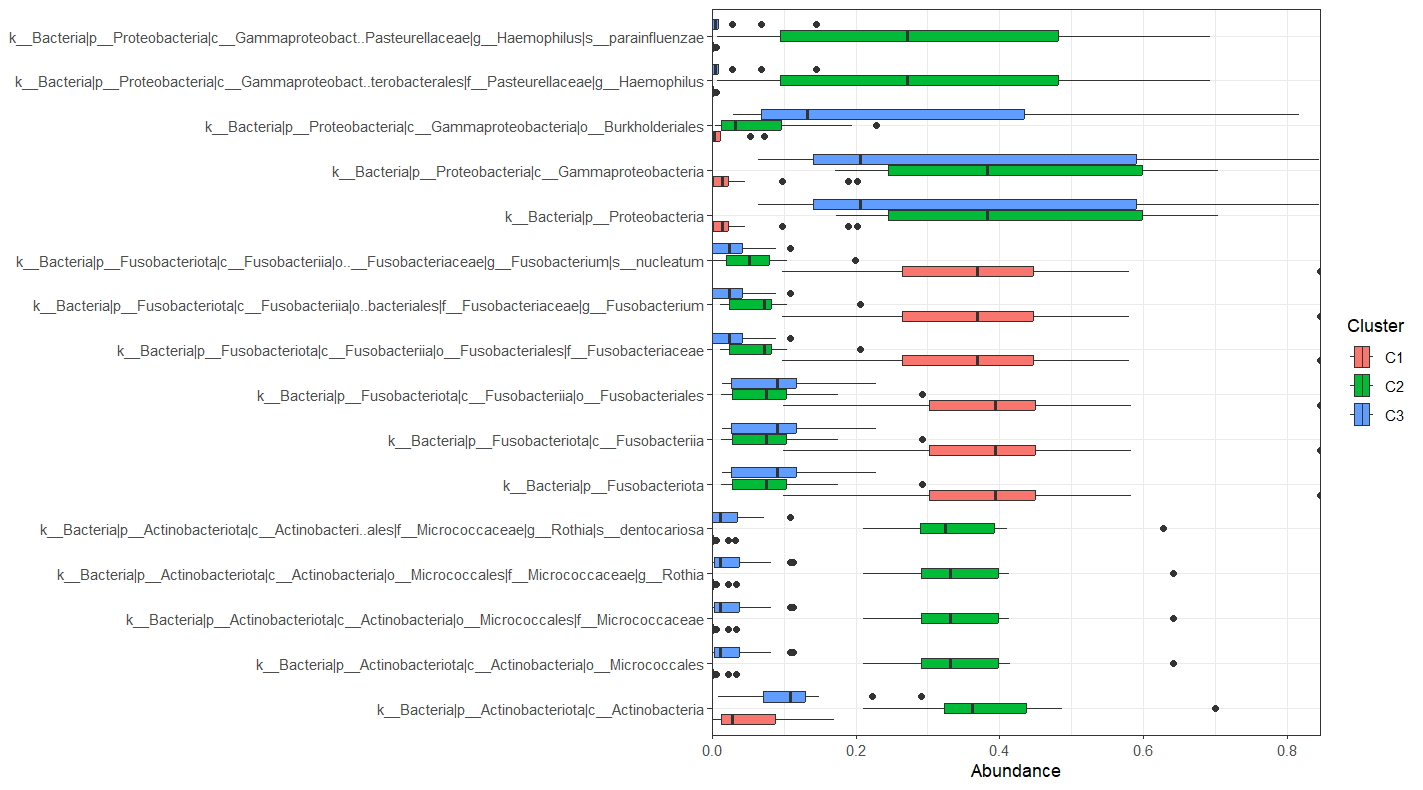


**Supplementary Figure S5** An abundance bar plot of differential taxa between the clusters C1, C2 and, C3. Differential abundance analysis was performed using the Kruskal-Wallis test (Bonferroni adjusted *p*-value cut-off value at 0.001) at all taxa levels.

**Supplementary Table S1** DADA2 processing results for 16S metagenomic amplicon sequencing data

| ID | input | filtered | denoisedF | denoisedR | merged | nonchim |
| --- | --- | --- | --- | --- | --- | --- |
| HI01 | 84122 | 61180 | 60995 | 61029 | 53288 | 52012 |
| HI01 | 94191 | 83000 | 81972 | 82346 | 75879 | 52938 |
| HI01 | 87152 | 76081 | 75594 | 75397 | 72908 | 60505 |
| HI02 | 88601 | 67045 | 65536 | 66140 | 59743 | 45349 |
| HI02 | 86627 | 74710 | 73277 | 74192 | 69253 | 60725 |
| HI02 | 77315 | 64883 | 61963 | 63093 | 53035 | 36574 |
| HI03 | 89131 | 69715 | 68102 | 68654 | 62059 | 43072 |
| HI03 | 87091 | 77569 | 76693 | 77092 | 70646 | 44045 |
| HI03 | 83532 | 73023 | 71001 | 72044 | 61095 | 39192 |
| HI04 | 98861 | 78677 | 77840 | 78250 | 73929 | 68246 |
| HI04 | 78122 | 61729 | 60175 | 60615 | 53951 | 38128 |
| HI04 | 61449 | 46571 | 45494 | 45981 | 41459 | 34572 |
| HI05 | 88590 | 70046 | 68590 | 69328 | 63629 | 52378 |
| HI05 | 94840 | 82627 | 80640 | 81149 | 74115 | 56772 |
| HI05 | 109577 | 95889 | 91761 | 93804 | 78449 | 49200 |
| HI06 | 105233 | 93569 | 92585 | 93033 | 87931 | 66599 |
| HI06 | 83552 | 74188 | 72940 | 73335 | 64686 | 41129 |
| HI06 | 104674 | 92507 | 91635 | 91941 | 86206 | 71223 |
| HI07 | 94715 | 81163 | 77895 | 79372 | 64742 | 36765 |
| HI07 | 107885 | 94694 | 90893 | 92900 | 79363 | 64882 |
| HI07 | 106034 | 95070 | 94734 | 94800 | 92064 | 87047 |
| PI01 | 96995 | 76827 | 76498 | 76624 | 75790 | 73564 |
| PI01 | 73257 | 64233 | 63634 | 63851 | 61527 | 52942 |
| PI01 | 92799 | 82253 | 81474 | 81647 | 76181 | 47729 |
| PI02 | 91126 | 72164 | 71158 | 71473 | 67912 | 59561 |
| PI02 | 96793 | 84431 | 79322 | 81799 | 64105 | 43552 |
| PI02 | 94537 | 80891 | 77111 | 78583 | 65951 | 49816 |
| PI03 | 74550 | 58278 | 57976 | 58151 | 57000 | 55118 |
| PI03 | 92323 | 82365 | 81245 | 81494 | 73211 | 42178 |
| PI03 | 102904 | 92194 | 90861 | 91394 | 82147 | 46315 |
| PI04 | 86306 | 69681 | 69382 | 69481 | 67378 | 66135 |
| PI04 | 75182 | 59368 | 59238 | 59260 | 56353 | 55195 |
| PI04 | 73964 | 63313 | 62920 | 63014 | 61339 | 59012 |
| PI05 | 65683 | 50825 | 48230 | 49320 | 41107 | 27016 |
| PI05 | 97123 | 85097 | 83322 | 84048 | 76727 | 51902 |
| PI05 | 88451 | 77193 | 73992 | 75613 | 64235 | 40149 |
| PI06 | 78368 | 61662 | 60315 | 60944 | 52035 | 35304 |
| PI06 | 111602 | 98294 | 96665 | 97207 | 87569 | 52870 |
| PI06 | 71127 | 62035 | 61132 | 61373 | 54501 | 27717 |
| PI07 | 83551 | 73303 | 68104 | 70720 | 54692 | 37010 |
| PI07 | 101146 | 88118 | 83393 | 85475 | 70794 | 47436 |
| PI07 | 94990 | 83893 | 79741 | 81646 | 68422 | 46413 |

**Supplementary Table S2** The 10 highest and lowest differentially abundant species between HI and PI groups at 0 months

|  | log2fold | *p*-value | Species |
| --- | --- | --- | --- |
| HI | -10.8272 | 0.000561 | *Campylobacter concisus* |
| HI | -8.63991 | 0.005029 | *Oribacterium sinus* |
| HI | -11.3603 | 0.008296 | *Actinomyces gerencseriae* |
| HI | -9.14925 | 0.010438 | *Fusobacterium periodonticum* |
| HI | -9.68373 | 0.020136 | *Capnocytophaga ochracea* |
| HI | -9.57996 | 0.020273 | *Stomatobaculum longum* |
| HI | -8.93079 | 0.02929 | *Veillonella atypica* |
| HI | -10.4533 | 0.032227 | *Streptococcus parasanguinis* |
| HI | -9.27509 | 0.034052 | *Porphyromonas pasteri* |
| HI | -2.63407 | 0.051331 | *Campylobacter gracilis* |
| PI | 8.524754 | 0.020665 | *Prevotella oris* |
| PI | 4.479977 | 0.029169 | *Porphyromonas gingivalis* |
| PI | 11.19872 | 0.032502 | *Neisseria bacilliformis* |
| PI | 2.796636 | 0.087897 | *Tannerella forsythia* |
| PI | 8.00158 | 0.120309 | *Akkermansia muciniphila* |
| PI | 4.894903 | 0.121643 | *Prevotella 7 baroniae* |
| PI | 2.957737 | 0.150133 | *Fretibacterium fastidiosum* |
| PI | 6.765253 | 0.162735 | *Lachnoanaerobaculum saburreum* |
| PI | 6.603018 | 0.167305 | *Leptotrichia buccalis* |
| PI | 3.701529 | 0.176163 | *Treponema denticola* |

**Supplementary Table S3** The 10 highest and lowest differentially abundant species between HI and PI groups at 3 months

|  | log2fold | *p*-value | Species |
| --- | --- | --- | --- |
| HI | -9.37837 | 0.030496 | *Actinomyces pacaensis* |
| HI | -8.11718 | 0.05335 | *Actinomyces oris* |
| HI | -4.40657 | 0.088138 | *Capnocytophaga granulosa* |
| HI | -6.2434 | 0.124786 | *Actinomyces georgiae* |
| HI | -6.0259 | 0.131482 | *Megasphaera micronuciformis* |
| HI | -4.77261 | 0.136599 | *Filifactor alocis* |
| HI | -5.72224 | 0.142017 | *Prevotella salivae* |
| HI | -4.9742 | 0.169987 | *Streptococcus parasanguinis* |
| HI | -4.80382 | 0.176761 | *Atopobium rimae* |
| HI | -3.70117 | 0.229411 | *Oribacterium parvum* |
| PI | 11.08233 | 0.001129 | *Phocaeicola abscessus* |
| PI | 10.34287 | 0.014691 | *Actinomyces gerencseriae* |
| PI | 5.317498 | 0.024114 | *Anaeroglobus geminatus* |
| PI | 8.568705 | 0.057729 | *Actinomyces israelii* |
| PI | 8.266879 | 0.064738 | *Prevotella micans* |
| PI | 7.781938 | 0.077563 | *Treponema parvum* |
| PI | 7.247002 | 0.093967 | *Prevotella 7 baroniae* |
| PI | 6.878163 | 0.105776 | *Olsenella uli* |
| PI | 6.828829 | 0.108817 | *Prevotella maculosa* |
| PI | 6.463222 | 0.12185 | *Prevotella 7 dentalis* |

**Supplementary Table S4** The 10 highest and lowest differentially abundant species between HI and PI groups at 6 months

|  | log2fold | *p*-value | Species |
| --- | --- | --- | --- |
| HI | -10.0928 | 0.01043 | *Veillonella rogosae* |
| HI | -9.47285 | 0.020114 | *Actinomyces oris* |
| HI | -8.64575 | 0.037652 | *Veillonella atypica* |
| HI | -8.44302 | 0.042208 | *Megasphaera micronuciformis* |
| HI | -5.33386 | 0.043029 | *Porphyromonas gingivalis* |
| HI | -4.24329 | 0.052448 | *Corynebacterium durum* |
| HI | -8.15314 | 0.052831 | *Desulfovibrio fairfieldensis* |
| HI | -7.54707 | 0.06945 | *Prevotella oulorum* |
| HI | -6.68816 | 0.104194 | *Scardovia wiggsiae* |
| HI | -6.386 | 0.113211 | *Prevotella 7 dentalis* |
| PI | 12.56569 | 0.000129 | *Actinomyces gerencseriae* |
| PI | 9.591417 | 0.01787 | *Streptococcus lactarius* |
| PI | 9.378456 | 0.021879 | *Gemella bergeri* |
| PI | 8.963665 | 0.031066 | *Capnocytophaga ochracea* |
| PI | 8.185374 | 0.04807 | *Johnsonella ignava* |
| PI | 6.558985 | 0.106308 | *Prevotella saccharolytica* |
| PI | 6.424029 | 0.110315 | *Treponema lecithinolyticum* |
| PI | 6.18854 | 0.117332 | *Leptotrichia hofstadii* |
| PI | 5.923371 | 0.125981 | *Catonella morbi* |
| PI | 5.915179 | 0.126545 | *Tistrella mobilis* |

**Supplementary Table S5** The 10 highest and lowest differentially abundant species between PI group at 0 months and PI group at 3 months

|  | log2fold | *p*-value | Species |
| --- | --- | --- | --- |
| 0-MONTH | -8.69506 | 0.001789 | *Porphyromonas gingivalis* |
| 0-MONTH | -10.5554 | 0.012905 | *Prevotella intermedia* |
| 0-MONTH | -9.42755 | 0.035405 | *Akkermansia muciniphila* |
| 0-MONTH | -9.31852 | 0.037872 | *Blautia glucerasea* |
| 0-MONTH | -6.8412 | 0.102876 | *Prevotella oulorum* |
| 0-MONTH | -6.36661 | 0.118612 | *Prevotella disiens* |
| 0-MONTH | -6.26974 | 0.121551 | *Actinomyces georgiae* |
| 0-MONTH | -4.11426 | 0.127075 | *Filifactor alocis* |
| 0-MONTH | -6.3352 | 0.142255 | *Prevotella 7 melaninogenica* |
| 0-MONTH | -5.63062 | 0.144967 | *Atopobium rimae* |
| 3-MONTH | 13.97849 | 0.000362 | *Actinomyces gerencseriae* |
| 3-MONTH | 10.34131 | 0.023734 | *Pseudopropionibacterium rubrum* |
| 3-MONTH | 8.358576 | 0.045106 | *Actinomyces israelii* |
| 3-MONTH | 8.311309 | 0.052831 | *Campylobacter concisus* |
| 3-MONTH | 4.196794 | 0.076251 | *Gemella morbillorum* |
| 3-MONTH | 8.022654 | 0.078161 | *Pseudoclavibacter faecalis* |
| 3-MONTH | 7.334487 | 0.085855 | *Campylobacter showae* |
| 3-MONTH | 6.1471 | 0.141931 | *Capnocytophaga ochracea* |
| 3-MONTH | 6.098204 | 0.143474 | *Oribacterium sinus* |
| 3-MONTH | 5.615474 | 0.14532 | *Stomatobaculum longum* |

**Supplementary Table S6** The 10 highest and lowest differentially abundant species between PI group at 0 months and PI group at 6 months

|  | log2fold | *p*-value | Species |
| --- | --- | --- | --- |
| 0-MONTH | -8.77546 | 0.00076 | *Porphyromonas gingivalis* |
| 0-MONTH | -9.84514 | 0.008573 | *Prevotella intermedia* |
| 0-MONTH | -9.57585 | 0.011529 | *Actinomyces oris* |
| 0-MONTH | -7.36441 | 0.013778 | *Neisseria bacilliformis* |
| 0-MONTH | -9.03728 | 0.020542 | *Desulfovibrio fairfieldensis* |
| 0-MONTH | -8.47996 | 0.033912 | *Akkermansia muciniphila* |
| 0-MONTH | -4.99711 | 0.035194 | *Filifactor alocis* |
| 0-MONTH | -8.37103 | 0.035914 | *Blautia glucerasea* |
| 0-MONTH | -4.12391 | 0.050998 | *Neisseria oralis* |
| 0-MONTH | -7.69545 | 0.051849 | *Veillonella rogosae* |
| 6-MONTH | 12.30005 | 0.000173 | *Actinomyces gerencseriae* |
| 6-MONTH | 5.188579 | 0.020091 | *Kingella oralis* |
| 6-MONTH | 8.698624 | 0.029191 | *Capnocytophaga ochracea* |
| 6-MONTH | 8.606035 | 0.031365 | *Fusobacterium periodonticum* |
| 6-MONTH | 7.482813 | 0.056327 | *Campylobacter showae* |
| 6-MONTH | 6.5109 | 0.092611 | *Campylobacter concisus* |
| 6-MONTH | 6.504882 | 0.092742 | *Actinomyces pacaensis* |
| 6-MONTH | 5.927788 | 0.109978 | *Leptotrichia hofstadii* |
| 6-MONTH | 5.654487 | 0.119095 | *Tistrella mobilis* |
| 6-MONTH | 5.488467 | 0.124747 | *Staphylococcus epidermidis* |

**Supplementary Table S7** The 10 highest and lowest differentially abundant species between PI group at 3 months and PI group at 6 months

|  | log2fold | *p*-value | Species |
| --- | --- | --- | --- |
| 3-MONTH | -4.96823 | 0.024072 | *Gemella morbillorum* |
| 3-MONTH | -4.54408 | 0.032502 | *Parvimonas micra* |
| 3-MONTH | -8.83351 | 0.040667 | *Desulfovibrio fairfieldensis* |
| 3-MONTH | -8.52737 | 0.046991 | *Actinomyces israelii* |
| 3-MONTH | -7.61188 | 0.072696 | *Pseudoclavibacter faecalis* |
| 3-MONTH | -6.24042 | 0.123058 | *Actinomyces oris* |
| 3-MONTH | -5.73072 | 0.141657 | *Sediminibacterium salmoneum* |
| 3-MONTH | -4.87974 | 0.149317 | *Pseudoramibacter alactolyticus* |
| 3-MONTH | -5.10881 | 0.16518 | *Prevotella maculosa* |
| 3-MONTH | -4.96469 | 0.171406 | *Veillonella rogosae* |
| 6-MONTH | 9.118874 | 0.028719 | *Atopobium rimae* |
| 6-MONTH | 4.590387 | 0.033512 | *Cardiobacterium hominis* |
| 6-MONTH | 5.868021 | 0.034254 | *Capnocytophaga granulosa* |
| 6-MONTH | 6.958167 | 0.085119 | *Actinomyces pacaensis* |
| 6-MONTH | 3.84376 | 0.091693 | *Leptotrichia hongkongensis* |
| 6-MONTH | 6.378355 | 0.103811 | *Leptotrichia hofstadii* |
| 6-MONTH | 6.181163 | 0.109727 | *Pseudopropionibacterium propionicum* |
| 6-MONTH | 6.104327 | 0.11219 | *Tistrella mobilis* |
| 6-MONTH | 5.937346 | 0.117378 | *Staphylococcus epidermidis* |
| 6-MONTH | 5.837486 | 0.120321 | *Treponema vincentii* |

**Supplementary Table S8** The 10 highest and lowest differentially abundant species between HI group at 0 months and HI group at 3 months

|  | log2fold | *p*-value | Species |
| --- | --- | --- | --- |
| 0-MONTH | -10.244 | 0.008706 | *Prevotella intermedia* |
| 0-MONTH | -8.35369 | 0.038845 | *Actinomyces gerencseriae* |
| 0-MONTH | -4.92681 | 0.056885 | *Peptostreptococcus stomatis* |
| 0-MONTH | -6.86496 | 0.059845 | *Fusobacterium periodonticum* |
| 0-MONTH | -3.68273 | 0.094463 | *Neisseria oralis* |
| 0-MONTH | -5.27108 | 0.110627 | *Leptotrichia massiliensis* |
| 0-MONTH | -6.47235 | 0.11217 | *Cryptobacterium curtum* |
| 0-MONTH | -5.49266 | 0.116598 | *Blautia glucerasea* |
| 0-MONTH | -6.12967 | 0.124353 | *Leptotrichia hofstadii* |
| 0-MONTH | -5.78782 | 0.134799 | *Fusobacterium necrophorum* |
| 3-MONTH | 8.759853 | 0.005749 | *Porphyromonas catoniae* |
| 3-MONTH | 10.1805 | 0.00791 | *Neisseria bacilliformis* |
| 3-MONTH | 9.429592 | 0.012405 | *Actinomyces pacaensis* |
| 3-MONTH | 9.379572 | 0.017261 | *Pseudopropionibacterium rubrum* |
| 3-MONTH | 7.264141 | 0.069942 | *Aggregatibacter segnis* |
| 3-MONTH | 6.780895 | 0.087037 | *Campylobacter curvus* |
| 3-MONTH | 6.697109 | 0.090123 | *Oribacterium asaccharolyticum* |
| 3-MONTH | 4.499327 | 0.091429 | *Capnocytophaga granulosa* |
| 3-MONTH | 6.66568 | 0.092447 | *Neisseria subflava* |
| 3-MONTH | 6.542512 | 0.097217 | *Atopobium rimae* |

**Supplementary Table S9** The 10 highest and lowest differentially abundant species between HI group at 0 months and HI group at 6 months

|  | log2fold | *p*-value | Species |
| --- | --- | --- | --- |
| 0-MONTH | -9.12773 | 0.026514 | *Prevotella intermedia* |
| 0-MONTH | -8.62676 | 0.027056 | *Actinomyces gerencseriae* |
| 0-MONTH | -8.74232 | 0.034648 | *Streptococcus lactarius* |
| 0-MONTH | -8.06074 | 0.035665 | *Bulleidia extructa* |
| 0-MONTH | -8.33618 | 0.043773 | *Streptococcus parasanguinis* |
| 0-MONTH | -7.47699 | 0.047778 | *Capnocytophaga ochracea* |
| 0-MONTH | -5.16245 | 0.050553 | *Neisseria oralis* |
| 0-MONTH | -7.16177 | 0.073196 | *Cryptobacterium curtum* |
| 0-MONTH | -6.50368 | 0.09682 | *Leptotrichia hofstadii* |
| 0-MONTH | -5.06906 | 0.10547 | *Shuttleworthia satelles* |
| 6-MONTH | 11.9748 | 0.001428 | *Campylobacter curvus* |
| 6-MONTH | 7.902005 | 0.026497 | *Pseudopropionibacterium rubrum* |
| 6-MONTH | 6.877591 | 0.028867 | *Oribacterium asaccharolyticum* |
| 6-MONTH | 4.616494 | 0.034268 | *Veillonella parvula* |
| 6-MONTH | 7.023179 | 0.038936 | *Prevotella oris* |
| 6-MONTH | 4.99176 | 0.047033 | *Leptotrichia hongkongensis* |
| 6-MONTH | 6.013308 | 0.057931 | *Actinomyces pacaensis* |
| 6-MONTH | 6.387382 | 0.07391 | *Lachnoanaerobaculum saburreum* |
| 6-MONTH | 5.000642 | 0.07881 | *Actinomyces israelii* |
| 6-MONTH | 6.071261 | 0.080625 | *Alloscardovia omnicolens* |

**Supplementary Table S10** The 10 highest and lowest differentially abundant species between HI group at 3 months and HI group at 6 months

|  | log2fold | *p*-value | Species |
| --- | --- | --- | --- |
| 3-MONTH | -10.6082 | 0.005204 | *Neisseria bacilliformis* |
| 3-MONTH | -7.10219 | 0.00694 | *Filifactor alocis* |
| 3-MONTH | -8.65365 | 0.007523 | *Porphyromonas catoniae* |
| 3-MONTH | -4.84288 | 0.027021 | *Capnocytophaga sputigena* |
| 3-MONTH | -7.68988 | 0.055763 | *Aggregatibacter segnis* |
| 3-MONTH | -7.35237 | 0.064884 | *Capnocytophaga ochracea* |
| 3-MONTH | -7.14848 | 0.071053 | *Johnsonella ignava* |
| 3-MONTH | -7.08994 | 0.073289 | *Neisseria subflava* |
| 3-MONTH | -6.06605 | 0.075813 | *Leptotrichia goodfellowii* |
| 3-MONTH | -6.20774 | 0.085452 | *Catonella morbi* |
| 6-MONTH | 6.455826 | 0.025115 | *Leptotrichia massiliensis* |
| 6-MONTH | 8.816274 | 0.040674 | *Prevotella oulorum* |
| 6-MONTH | 7.12306 | 0.090597 | *Treponema parvum* |
| 6-MONTH | 7.073588 | 0.092509 | *Scardovia wiggsiae* |
| 6-MONTH | 6.766263 | 0.105175 | *Prevotella 7 dentalis* |
| 6-MONTH | 6.264606 | 0.113165 | *Actinomyces israelii* |
| 6-MONTH | 3.688786 | 0.127016 | *Porphyromonas gingivalis* |
| 6-MONTH | 5.967336 | 0.129577 | *Prevotella maculosa* |
| 6-MONTH | 5.532195 | 0.137643 | *Pseudopropionibacterium propionicum* |
| 6-MONTH | 5.572037 | 0.14335 | *Alloscardovia omnicolens* |

**Supplementary Table S11** Significant pathways identified from the comparison between HI and PI groups at baseline (0 months)

| Pathway | Biological relevance | *p*-value | Mean Abundance PI (%) | Mean Abundance HI (%) |
| --- | --- | --- | --- | --- |
| pyrimidine deoxyribonucleotides de novo biosynthesis | Potentially associated with virulence factor production (1). | 0.037 | 0.521 | 0.663 |
| 4-deoxy-L-threo-hex-4-enopyranuronate degradation | Involved in the production of short-chain fatty acids (SCFAs) (2). | 0.014 | 0.046 | 0.013 |
| adenosine nucleotides degradation II | Linked to host–microbe interactions through modulation of energy metabolism and cellular signaling (3). | 0.026 | 0.163 | 0.067 |
| UDP-2,3-diacetamido-2,3-dideoxy-alpha-D- mannuronate biosynthesis | Involvement of the Wbp pathway in the biosynthesis of A-LPS (4). | 0.018 | 0.043 | 0.011 |
| 4-aminobutanoate degradation V | Related with butyrate production (5). | 0.009 | 0.168 | 0.045 |
| superpathway of UDP-N-acetylglucosamine-derived O-antigen building blocks biosynthesis | Related to lipopolysaccharide (LPS) biosynthesis (6). | 0.011 | 0.093 | 0 |
| myo-inositol degradation I | Involved in bacterial biofilm formation (7). | 0.049 | 0.010 | 0.052 |

**Supplementary Table S12** Summary of surgical interventions

| Patient No. | Surgical Technique | Membrane Type | Graft Material |
| --- | --- | --- | --- |
| 1 | Regenerative surgical therapy | Jason® membrane | Straumann® xenograft |
| 2 | Regenerative surgical therapy | Jason® membrane | Straumann® xenograft |
| 3 | Regenerative surgical therapy | Jason® membrane | Straumann® xenograft |
| 4 | Regenerative surgical therapy | Jason® membrane | Straumann® xenograft |
| 5 | Regenerative surgical therapy | Jason® membrane | Straumann® xenograft |
| 6 | Access flap surgery | — | — |
| 7 | Access flap surgery | — | — |

**Supplementary Table S13** Cluster membership of samples

| Sample | Site | Cluster | Patient | Time Point (months) |
| --- | --- | --- | --- | --- |
| AK14_0M | AK14 | C1 | AK | 0 |
| AK16_0M | AK16 | C1 | AK | 0 |
| BS36_0M | BS36 | C1 | BS | 0 |
| BS46_0M | BS46 | C1 | BS | 0 |
| PP12_0M | PP12 | C1 | PP | 0 |
| SK35_0M | SK35 | C1 | SK | 0 |
| SP26_0M | SP26 | C1 | SP | 0 |
| SP47_0M | SP47 | C1 | SP | 0 |
| PT17_0M | PT17 | C2 | PT | 0 |
| PT45_0M | PT45 | C2 | PT | 0 |
| SK14_0M | SK14 | C2 | SK | 0 |
| PB14_0M | PB14 | C3 | PB | 0 |
| PB27_0M | PB27 | C3 | PB | 0 |
| PP23_0M | PP23 | C3 | PP | 0 |
| AK14_3M | AK14 | C1 | AK | 3 |
| AK16_3M | AK16 | C1 | AK | 3 |
| PP12_3M | PP12 | C1 | PP | 3 |
| SP26_3M | SP26 | C1 | SP | 3 |
| SP47_3M | SP47 | C1 | SP | 3 |
| PT45_3M | PT45 | C2 | PT | 3 |
| SK14_3M | SK14 | C2 | SK | 3 |
| SK35_3M | SK35 | C2 | SK | 3 |
| BS36_3M | BS36 | C3 | BS | 3 |
| BS46_3M | BS46 | C3 | BS | 3 |
| PB14_3M | PB14 | C3 | PB | 3 |
| PB27_3M | PB27 | C3 | PB | 3 |
| PP23_3M | PP23 | C3 | PP | 3 |
| PT17_3M | PT17 | C3 | PT | 3 |
| SK35_6M | SK35 | C2 | SK | 6 |
| AK14_6M | AK14 | C1 | AK | 6 |
| PP12_6M | PP12 | C1 | PP | 6 |
| PP23_6M | PP23 | C1 | PP | 6 |
| SP26_6M | SP26 | C1 | SP | 6 |
| SP47_6M | SP47 | C1 | SP | 6 |
| PB14_6M | PB14 | C2 | PB | 6 |
| PT45_6M | PT45 | C2 | PT | 6 |
| SK14_6M | SK14 | C2 | SK | 6 |
| AK16_6M | AK16 | C3 | AK | 6 |
| BS36_6M | BS36 | C3 | BS | 6 |
| BS46_6M | BS46 | C3 | BS | 6 |
| PB27_6M | PB27 | C3 | PB | 6 |
| PT17_6M | PT17 | C3 | PT | 6 |

References

1. Goncheva MI, Chin D, Heinrichs DE. Nucleotide biosynthesis: the base of bacterial pathogenesis. Trends Microbiol. 2022;30(8):793-804.

2. Zhang F, Wan Y, Zuo T, Yeoh YK, Liu Q, Zhang L, et al. Prolonged Impairment of Short-Chain Fatty Acid and L-Isoleucine Biosynthesis in Gut Microbiome in Patients With COVID-19. Gastroenterology. 2022;162(2):548-61 e4.

3. Miller SG, Hafen PS, Brault JJ. Increased Adenine Nucleotide Degradation in Skeletal Muscle Atrophy. Int J Mol Sci. 2019;21(1).

4. Shoji M, Sato K, Yukitake H, Naito M, Nakayama K. Involvement of the Wbp pathway in the biosynthesis of Porphyromonas gingivalis lipopolysaccharide with anionic polysaccharide. Sci Rep. 2014;4:5056.

5. Singh V, Lee G, Son H, Koh H, Kim ES, Unno T, et al. Butyrate producers, "The Sentinel of Gut": Their intestinal significance with and beyond butyrate, and prospective use as microbial therapeutics. Front Microbiol. 2022;13:1103836.

6. Toh KY, Toh TS, Chua KP, Rajakumar P, Lee JWJ, Chong CW. Identification of age-associated microbial changes via long-read 16S sequencing. Gut Pathog. 2024;16(1):56.

7. O'Donovan CM, Madigan SM, Garcia-Perez I, Rankin A, O OS, Cotter PD. Distinct microbiome composition and metabolome exists across subgroups of elite Irish athletes. J Sci Med Sport. 2020;23(1):63-8.
